# Amplitude and Temporal Dynamics of Motion Sickness

**DOI:** 10.3389/fnsys.2022.866503

**Published:** 2022-05-09

**Authors:** Tugrul Irmak, Varun Kotian, Riender Happee, Ksander N. de Winkel, Daan M. Pool

**Affiliations:** ^1^Cognitive Robotics Department, Delft University of Technology, Delft, Netherlands; ^2^Control and Simulation Department, Delft University of Technology, Delft, Netherlands

**Keywords:** motion sickness, mathematical modeling, sensory conflict, stimulus amplitude, power-law scaling, forecasting

## Abstract

The relationship between the amplitude of motion and the accumulation of motion sickness in time is unclear. Here, we investigated this relationship at the individual and group level. Seventeen participants were exposed to four oscillatory motion stimuli, in four separate sessions, separated by at least 1 week to prevent habituation. Motion amplitude was varied between sessions at either 1, 1.5, 2, or 2.5 ms^−2^. Time evolution was evaluated within sessions applying: an initial motion phase for up to 60 min, a 10-min rest, a second motion phase up to 30 min to quantify hypersensitivity and lastly, a 5-min rest. At both the individual and the group level, motion sickness severity (MISC) increased linearly with respect to acceleration amplitude. To analyze the evolution of sickness over time, we evaluated three variations of the Oman model of nausea. We found that the slow (502 s) and fast (66.2 s) time constants of motion sickness were independent of motion amplitude, but varied considerably between individuals (slow STD = 838 s; fast STD = 79.4 s). We also found that the Oman model with output scaling following a power law with an exponent of 0.4 described our data much better as compared to the exponent of 2 proposed by Oman. Lastly, we showed that the sickness forecasting accuracy of the Oman model depended significantly on whether the participants had divergent or convergent sickness dynamics. These findings have methodological implications for pre-experiment participant screening, as well as online tuning of automated vehicle algorithms based on sickness susceptibility.

## 1. Introduction

Motion sickness is a syndrome that arises as a consequence of a wide range of self-motion and orientation cues. It is characterized by symptoms of sweating, headache, dizziness, stomach awareness, where these symptoms usually grow in severity until nausea, retching and ultimately vomiting occurs (Bertolini and Straumann, 2016). The fact that adverse motions may, in a wide range of species (Wang and Chinn, 1956; Wassersug et al., 1993; Bauerle et al., 2004; Hickman et al., 2008), cause a diverse set of symptoms is peculiar.

Therefore, the etiology of motion sickness remains an active area of scientific inquiry. There are two main theories of motion sickness, these are the “Sensory Conflict” (Reason, 1978; Oman, 1982) theory and the “Postural Instability” theory (Riccio and Stoffregen, 1991). The most developed mathematical models and tools exist for the sensory conflict theory (Bos and Bles, 1998; Khalid et al., 2011; Wada, 2021). Therefore, this paper will study motion sickness through the concepts of state estimation and sensory conflict, and will not cover the postural instability theory nor attempt to evaluate postural precursors to motion sickness.

The sensory conflict theory (Reason, 1978) argues that motion sickness is mainly due to a conflict between the sensed sensory signals and the sensory signals expected by the brain. These expectations originate from an internal model, which takes the form of a neural store. The conflict leads to adaptation of the internal model. In the formulation of Oman (1982) this conceptual model is likened to a Luenberger Observer (LO). The LO has an internal model of the system (body) and sensor dynamics. Due to the imperfect and noisy nature of the sensory signals, one cannot use the sensor measurements directly. Instead, the true states of the system must be observed (estimated) by integrating sensory information using an internal model of the system itself. Indeed, there is strong neuronal evidence for the use of internal models for state estimation (Merfeld et al., 1999; Angelaki et al., 2004; Laurens et al., 2013; Oman and Cullen, 2014). To quantify estimation accuracy, the central state estimates are passed through an internal model of sensory dynamics and compared with the actual sensory signals. The resulting error is the estimation error, or the sensory-expectancy conflict. It is hypothesized that the magnitude of the conflict and the duration of exposure then leads to the subsequent symptoms of motion sickness.

There are practical implications that come with a firm understanding of the relationship between the magnitude of sensory conflict and motion sickness accumulation. Firstly, such knowledge allows us to better generalize motion sickness predictions to mixed acceleration environments that are ubiquitous to vehicular transport (Feng et al., 2017). Such predictions may then be used as an objective function to minimize sickening vehicle motions. Secondly, a functional model will allow for the development of control algorithms that can automatically adjust the amplitude of sickening simulator motions such that participants track a desired sickness trajectory. Currently, in experimental studies, researchers must fix their stimulus beforehand and hope that participants do become sick, but do not terminate the experiment prematurely. Active control will allow for setting the desired level and variance of motion sickness, which will increase the statistical quality of data collected. Lastly, a predictive model of sickness accumulation will allow for tuning of automated vehicle algorithms to the susceptibility level of the individual passenger, whilst also allowing prescreening of participants for a desired level of susceptibility. To allow for these novel methods and technologies, the mathematical process that links sensory conflict to the time evolution of motion sickness must be elucidated.

For simple motions, such as single degree-of-freedom vertical or horizontal accelerations, the conflict vector is assumed to be proportional to the acceleration stimulus itself. There is literature on the relationship between the acceleration magnitude (a proxy for the magnitude of the conflict) and group-level responses to sickness. Lawther and Griffin (1988), for instance, show a linear relationship between the amplitude of vertical accelerations on ships and motion sickness incidence (MSI), which is the percentage of people who vomited during the exposure. Likewise, using the more sensitive metric of mean subjective illness score, they also observed a strongly linear relationship between acceleration amplitude and sickness. However, their tested acceleration amplitudes were only in the range of 0–0.7 ms^−2^, which covers a small linearisable part of the complete, possibly nonlinear sickness amplitude dynamics. Indeed, looking at the data of O'Hanlon and McCauley (1973), in the range of 0.25–3.9 ms^−2^ there seems to be a sigmoidal relationship between acceleration amplitude and MSI. The subjective vertical model developed by Bos and Bles (1998) captures this sigmoidal relationship by first rectifying the conflict vector, and then input scaling it with a non-linear Hill-function. The resulting scaled conflict is then integrated with a second-order system, to match the MSI observations of O'Hanlon and McCauley (1973).

The approach of combining sensory conflict and accumulation models is unique because it clearly discriminates between conflict *generation*, which is a by-product of spatial orientation and state estimation, and conflict *integration*, which leads to motion sickness. There are two shortcomings in this approach. Firstly, at the practical level, the motion sickness prediction is made using motion sickness incidence (MSI), defined as the percentage of people that have vomited. This misses the finer increments in symptom development that precede vomiting, which are more relevant for most practical applications of motion sickness modeling. Secondly, the approach conflates the internal dynamics that lead to sickness at an individual-level with the averaged group-level dynamics. For a physiologically valid model of motion sickness, the final sickness predictions should map to individual ratings, not group-averaged ones.

An individual-level model of the temporal dynamics of motion sickness was developed by Oman (1990). This model is also uniquely able to describe the phenomenon of “hypersensitivity”, which is an essential part of sickness development over time. Hypersensitivity is characterized by the fact that after exposure to sickening motions, any further exposure to sickening motions leads to a more rapid rise in sickness than in the initial exposure (Golding et al., 1997). Modeling hypersensitivity is particularly relevant for automated driving, as sickening motions are usually separated by long durations of rest (i.e., at the traffic lights). In our previous work, the Oman model was validated in the context of motion sickness generated by slalom maneuvers performed by a car at 0.2 Hz with a lateral acceleration of 4 ms^−2^, for up to 30 min (Irmak et al., 2020). Here, it was seen that the model provided a good fit to subjective sickness scores as measured on the MIsery rating SCale (MISC) (Bos et al., 2010). Moreover, using the Oman model, parameters governing the trajectory of motion sickness could be used to predict individual responses in re-exposure to the same paradigm. This indicated a high degree of intra-individual repeatability in sickness dynamics. In this previous experiment, we only used an acceleration of a single magnitude. However, in traffic, humans generally encounter mixed acceleration stimuli. The original form of Oman (1990)'s model predicts the end level of sickness to be a quartic of the input acceleration amplitude. This is because of the model's “slow” path acting as a gain on its “fast” path and the output power scaling, *p*_*o*_ shown in **Figure 2**, being set to 2. It is, however, not clear whether this proposed amplitude relationship is correct. Nor is it clear whether the Oman model can generalize to fit sickness for different acceleration inputs, or whether its parameters must be refitted on a case-by-case basis.

In the present study, we assessed the relationship between conflict magnitude, using acceleration stimulus amplitude as a proxy, and the temporal dynamics of motion sickness symptoms at the individual-level. We did this by exposing 17 participants to sinusoidal fore-aft motions of four different acceleration amplitudes. The experiment was performed in the SIMONA Research Simulator at the Aerospace Engineering faculty of TU Delft (Stroosma et al., 2003; Berkouwer et al., 2005). In the subsequent analyses, we first confirm previous literature findings for the relationship between acceleration amplitude and group-level responses. Uniquely, we show that motion sickness at an individual-level for different stimulus amplitudes can be modeled adequately with individually varying Oman model time constants that are independent of motion amplitude, but using the same group-level averaged power law scaling at the output for all individuals. Moreover, we show that the Oman model in its current form can forecast the future evolution of motion sickness, but the accuracy of the forecasting is dependent on the qualitative form of individuals' sickness dynamics. This has important consequences for prescreening of participants for motion sickness experiments, and tuning of automated driving algorithms to individual passengers.

## 2. Methods

### 2.1. Participants

In total, 17 participants completed this study (mean age: 25.3 years, STD: 2.6 years; 2 female, 15 male). The 17 participants had a mean motion sickness susceptibility questionnaire short form (MSSQ-Short, Golding, 2006) score of 16.2 (STD = 10.1) indicating that they had above average susceptibility, corresponding to the 65th percentile.

### 2.2. Apparatus

**Figure 1:**
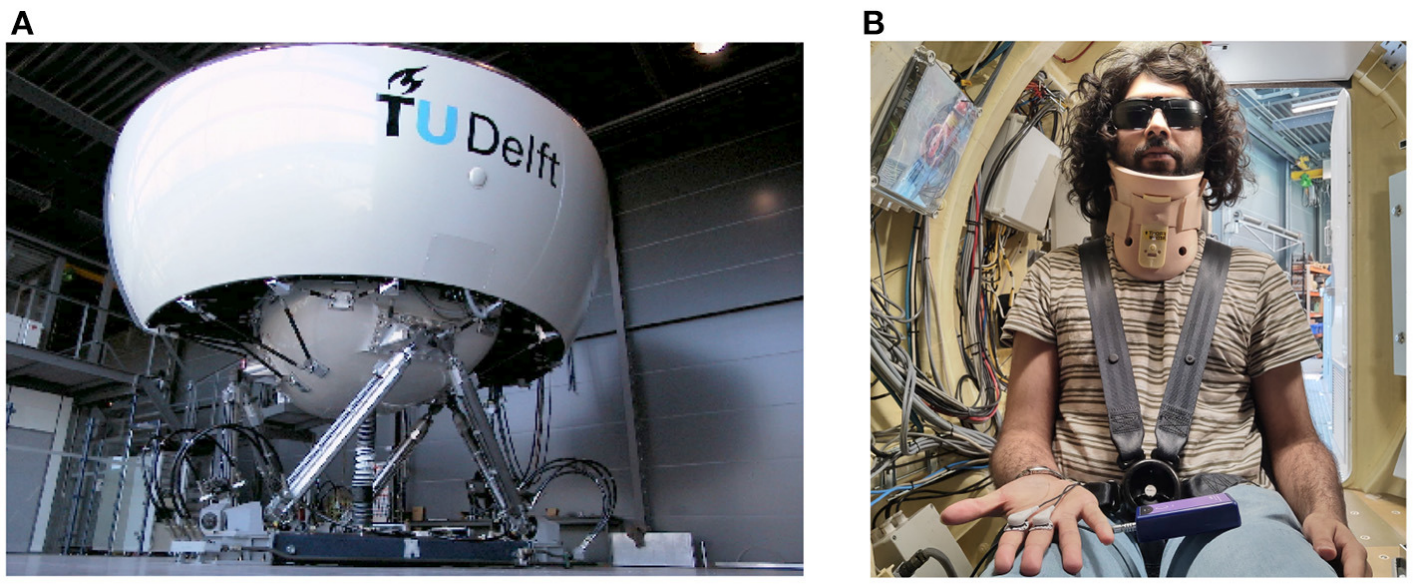
Experimental setup. **(A)** is the SIMONA Research Simulator used in the experiment to elicit motion sickness via fore-aft sinusoidal motions of differing amplitudes. **(B)** shows the second author as a participant in the experiment, wearing a 5-point safety harness, a neck restraint, and blackened glasses.

The experiment was performed in the SIMONA Research Simulator at TU Delft (Figure 1). The simulator has a six degree-of-freedom hydraulic hexapod motion system, which can provide a maximum displacement of 1.12 m, a maximum velocity of 0.9 ms^−1^ and a maximum acceleration of 13 ms^−2^ (Stroosma et al., 2003; Berkouwer et al., 2005). The participants were placed inside a closed cabin, within which they were seated and secured using a five point harness. To prevent unwanted head movements, their head was supported with a neck restraint. To remove any visual cues, they wore blackened goggles and the cabin lights were turned off (see Figure 1). Continuous communication with the experimenter was possible via an intercom system.

### 2.3. Task

Each condition was tested on participants with a rest of at least 1 week (mean: 30.6 days, STD: 20.6 days) in between any two test conditions. In these sessions, participants were subjected to sinusoidal fore-aft motions at a frequency of 0.3 Hz. The amplitude of the accelerations used were; 1, 1.5, 2, and 2.5 ms^−2^. The choice of the highest acceleration was constrained by the maximum possible simulator velocity of 0.9 ms^−1^. The choice of the frequency was based on the highest frequency observed for which the population incidence of sickness does not decrease (Golding and Markey, 1996; Golding et al., 1997; Irmak et al., 2020).

In each session, participants underwent two motion exposures. The first exposure lasted for 60 min, or until the participant reached a MISC of 6. They were then permitted 10 min rest, after which the second exposure lasted for 30 min, or until they reached a MISC of 6. After this, they first rested for 5 min in the simulator, and then for as long as they desired to in the staging room. At the beginning and end of each motion, the motions were faded in and out with a linearly increasing and decreasing amplitude from zero to the level specified over a 10-s period.

Each session only tested one amplitude of the range of acceleration stimuli. Due to time limitations and a desire to sample as broad a range of amplitudes as possible, conditions were not repeated. This is justified by good trial-to-trial repeatability found previously in measured motion sickness responses (Miller II and Graybiel, 1969; Irmak et al., 2020). The order in which each amplitude was experienced was balanced between participants using a Latin square. This prevented confounding effects of habituation between the different amplitudes.

### 2.4. Quantifying Sickness

Participants were instructed to report their sickness on the 11-point MISC scale (Bos et al., 2010). The MISC scale is anchored to specific motion sickness symptoms: 0 is no symptoms, 1 is uneasiness, 2, 3, 4, 5 represent increasing severity of non-nausea symptoms from vague to severe, 6 is mild nausea, 7 is moderate nausea, 8 is severe nausea with 9 and 10 being retching and vomiting, respectively. The MISC is useful because the ratings are directly linked to symptoms, which is not the case with other scales such as the Fast Motion Sickness Scale (FMS) (Keshavarz and Hecht, 2011) and the Magnitude Estimate scale (Bock and Oman, 1982). Having a non-anchored scale would make the ultimate aim of minimizing of sickness predictions with respect to vehicle motions infeasible. It has been reported by Reuten et al. (2021) that there is a clear non-monotonic relationship between a MISC level of 5 and 6 in terms of the feelings of unpleasantness that are often used to characterize the sickness response. However, recently (de Winkel et al., 2022) have demonstrated that this observed break from monotonicity was semantic in nature. The discomfort associated with each level of the MISC, as it was used to express motion sickness during exposure to a sickening stimulus, was found to increase monotonously and the MISC could be characterized by a power law of exponent 1.206 (de Winkel et al., 2022). All together, these considerations were deemed sufficient to warrant using the MISC directly for our modeling work.

Every 30 s, a 1 kHz beep was played over the simulator intercom to prompt the participant to verbally state their MISC level. In addition to this prompted response, participants were told that they could voluntarily give a MISC report whenever they thought it changed substantially since the last response was requested. Responses were recorded on audio and transcribed after the experiment session by the experimenter. The audio recordings were voice activated and recorded only for the duration the participant was speaking. Each MISC rating given by the participant was time stamped to the start of the audio sample.

#### 2.4.1. Drop-Out Rate and MISC Rate

To quantify the dynamics of sickness with respect to acceleration amplitude, the severity of sickness must be specified. To this end, we used the MISC rate and the drop-out rate. The MISC rate is defined as the MISC rating at the end of motion exposure, divided by the time in minutes to this end. Whereas, the drop-out rate is simply defined as the percentage of participants that have prematurely terminated a motion exposure.

### 2.5. Sickness Model

**Figure 2:**
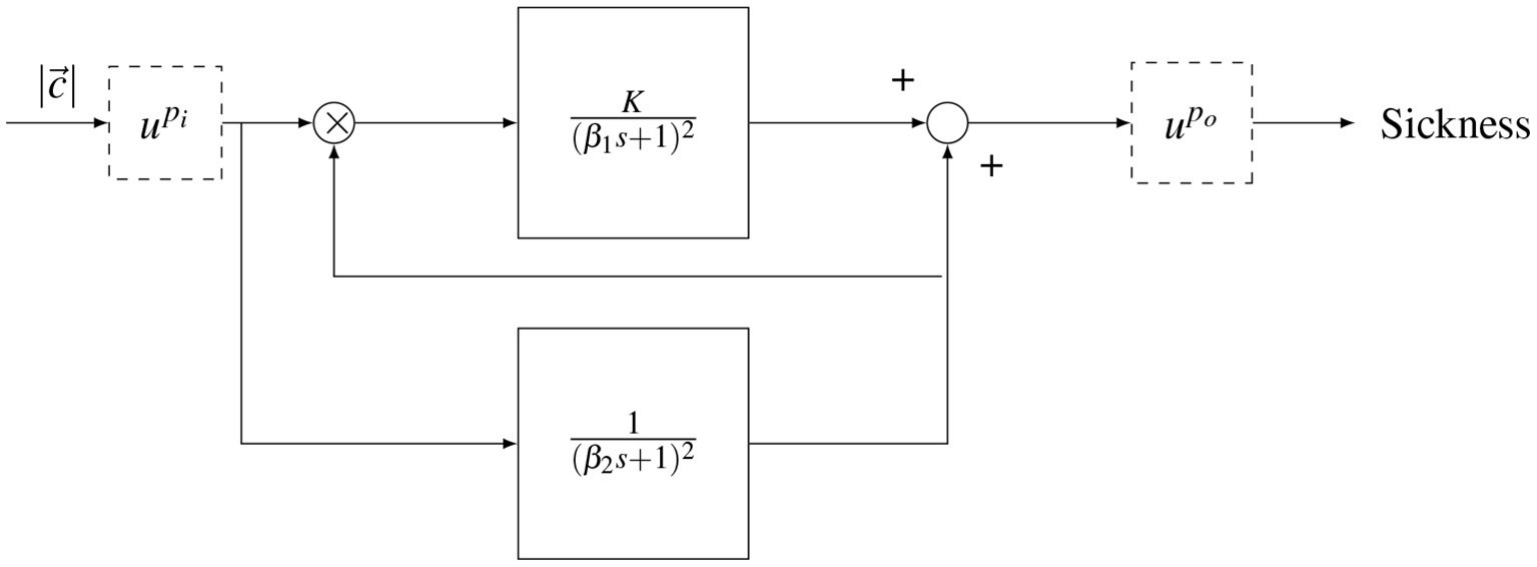
Oman's model of motion sickness development in time. The rectified conflict signal |*c*| is fed in to the model. There is a fast (upper) path and a slow (lower) path. The slow path multiplies with the conflict as the gain of the fast path. Both systems are second order with repeated poles. The fast and slow path are then summed. The model has either an input power scaling or an output power scaling given by $u^{p_{i}}$ and $u^{p_{o}}$, respectively.

The sickness accumulation model in this study is the Oman (1990) model shown in Figure 2. Here, the input to the model is the magnitude of the rectified sensory-expectancy conflict. In advanced sensory integration models, the sensory conflict is a product of the state estimation/motion perception process (Clark et al., 2019; Wada, 2021). In this experiment, the motions encountered were simple fore-aft accelerations and the sensory conflict was therefore assumed to be proportional to the acceleration stimulus itself.

The output of the model is a generic sickness level, which may be quantified with a sickness rating scale such as the MISC. In the model, there is a “fast” path and a “slow” path. The fast path is given by a repeated root second-order system with a time constant β_1_. The slow path is given by a repeated root second-order system with a time constant β_2_. The slow path controls the gain on the fast path. The existence of the two paths, rather than one standard path as in the subjective vertical model (SVM), enables (Oman, 1990)'s model to describe the phenomenon of hypersensitivity.

The original form of the Oman model has an output scaling ($u^{p_{o}}$), where the sum of the fast and slow paths are raised to the power of 2 (*p*_*o*_ = 2), a choice which has, to the best of our knowledge, not been validated. An alternative is an input scaling, which represents a direct sensitivity relationship between sensory-expectancy conflict and motion sickness at the input level, as proposed in Bos and Bles (1998). In this study, both input and output scaling were explored, but as output scaling provided a better fit to the data, this is the model form reported in the results. Nevertheless, we discuss the effect of input and output scaling in the discussion section.

All poles of the Oman model are negative, meaning it has a stable response that eventually converges to a steady-state level of sickness *MISC*_*ss*_. For a step input of amplitude *A*, the effect of output scaling on the model output, is given by the equation


(1) $$\begin{array}{r} {\text{MISC}}_{ss}={\left(KA^{2}+A\right)}^{p_{o}} \end{array}$$

where *K* is the gain of the fast path and *p*_*o*_ is the output power scaling of the conflict amplitude.

#### 2.5.1. Error Metric

The formulation of the Oman model considered in this study has four parameters. These are the fast and slow path time constants β_1_, β_2_, the gain *K* and the output power scaling exponent *p*_*o*_.

The error metric used for the optimization was the mean absolute error (MAE), which is given by the equation:


(2) $$\begin{array}{r} \text{MAE}=\frac{\Sigma_{t=1}^{n}|F_{t}-A_{t}|}{n} \end{array}$$

For each iteration of the optimization an error is calculated using the predicted MISC ratings *F*_*t*_ and the measured *A*_*t*_ ratings. The MAE is not scaled, this means it fits the higher MISC ratings more faithfully than the lower scores. Moreover, it is easy to interpret, as the MAE directly quantifies the average absolute deviation from the observation.

#### 2.5.2. Optimization Procedure

Three variations of Oman's model were fitted to the individual participants' data:

1. **Session Fit, Unit Power:** As a baseline for how well the model could feasibly fit the sickness profile, but also to assess how the model parameters may vary between conditions, each session was fitted separately. This means that the time constants β_1_, β_2_ and the gain *K* were fitted for each individual session, and thus stimulus amplitude. The power was assumed to be unity, i.e., *p*_*o*_ = 1, and fixed for all fittings. The optimization was performed using the MATLAB fmincon function. Due to the presence of local minima, this was done using 10 multi-starts.
2. **Joint Fit, Individual-Level Power:** The first model does not have a generalizable amplitude relationship from which one can make predictions across acceleration levels. For this reason, the sickness to amplitude relationship is assumed to be an idiosyncratic property of the individual, and so another model was fitted where the power law term was allowed to vary between participants. The fits were done jointly for all conditions for a given individual, meaning that both the time constants (β_1_ and β_2_), the gain (*K*) and the power law (*p*_*o*_) terms did not vary within an individual between the different conditions, but did vary between individuals. The optimization was performed using fmincon with 10 multi-starts.
3. **Joint Fit, Group-Level Power:** To assess whether an individual power law was needed to adequately capture the sickness observations, or whether a group-level power law metric is sufficient, the model was fitted with a power law *p*_*o*_ term that was fixed between participants. The fits were done jointly for all conditions for a given individual, meaning that both the time constants (β_1_ and β_2_) and the gain (*K*) did not vary within an individual between the conditions. The optimization was done using fmincon with 10 multi-starts.

### 2.6. Statistical Analysis

#### 2.6.1. AICc

Models with more free parameters generally give better fits to experimental data. To assess the significance of such additional model parameters, we used the corrected Akaike Information Criterion (AICc). This is a measure of model fit that is based on the likelihood of the data given the model, whilst including a penalty term for the number of parameters. It is a corrected form of the AIC where the parameter penalty scales quadratically, but approaches the AIC when the number of observations, *n* is many times larger than *k*^2^. Fab (2014) explains how to interpret the absolute value of differences in the AICc between the models, in terms of strength of evidence. According to these rules of thumb, absolute differences in the indices >2, >6, and >10 provide positive, strong, and decisive evidence, respectively, in favor of the model with a lower AICc value.

#### 2.6.2. Friedman Test

As our metrics do not satisfy the assumptions required for parametric testing, the Friedman test was used for statistical comparisons between different amplitude conditions. The Friedman test is a non-parametric test analogous to the parametric repeated-measures ANOVA. The significance level is reported in much the same way as in an ANOVA, where a *p*-value that is less or equal to 0.05 is taken as indication of a statistically significant result.

#### 2.6.3. Logrank test

Logrank test is a hypothesis test used to compare the survival distribution of two samples. In this study, it was used to compute a pairwise comparison between the termination curves of different motion conditions.

## 3. Results

### 3.1. Group-Level Observations

**Figure 3:**
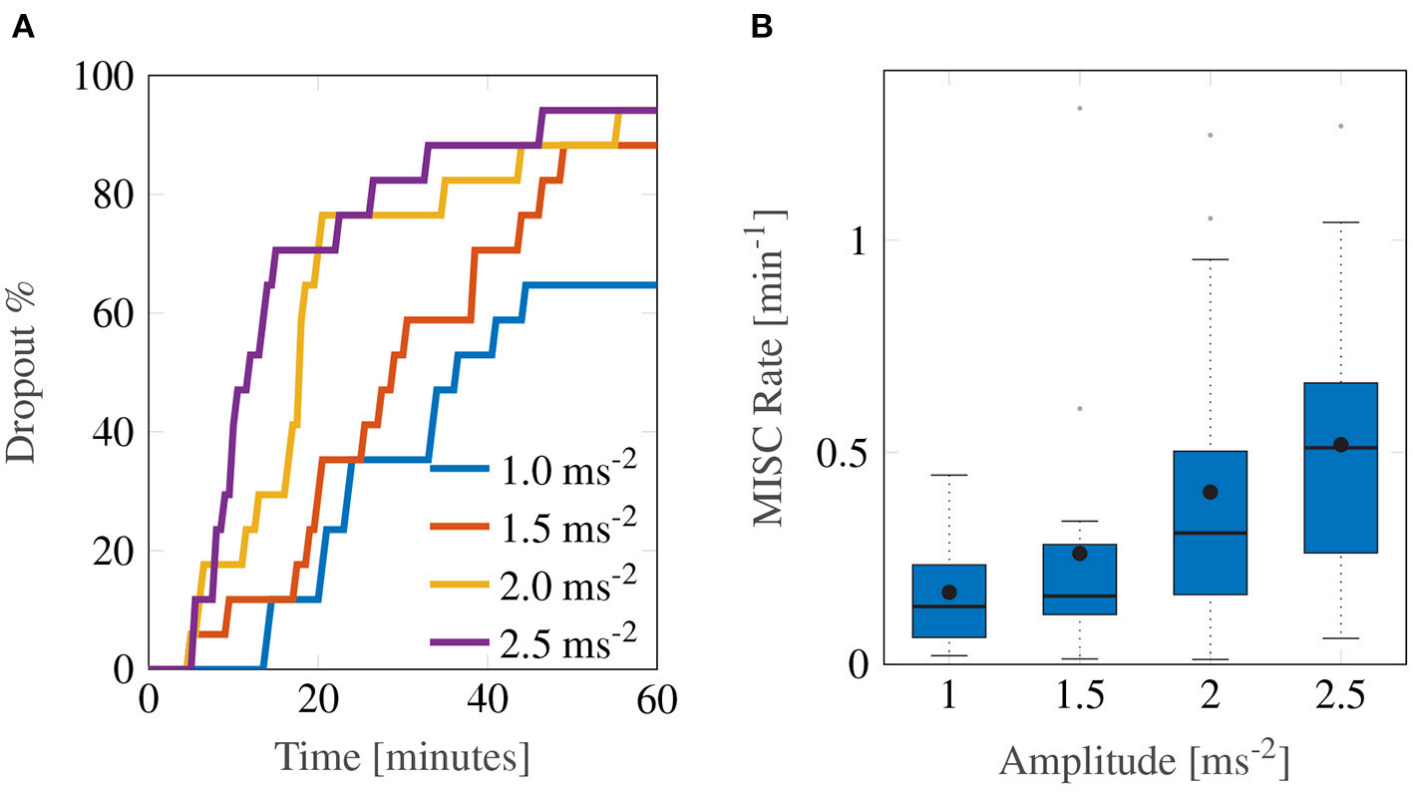
Main group-averaged results for the first motion exposure. **(A)** indicates the early termination rate where participants reached a MISC level of 6 prior to the 60-min mark in first motion exposure. **(B)** shows the median MISC Rate (as the solid black line inside blue shaded box) the mean MISC Rate (black circle) and the 25th and 75th percentiles (bounds of the box).

The experiment proved to be very sickening. Figure 3 shows the group-level results for all 17 participants over the first 60-min motion exposure. The dropout rate for all four conditions is shown in Figure 3A and was high, whereby the three highest amplitudes—2.5, 2.0, and 1.5 ms^−2^—had similar dropout rates after 60 min of approximately 94%. The lowest amplitude setting had an appreciably lower dropout rate of 64.7%. Using a logrank test between the amplitudes 1 and 1.5 ms^−2^, 1.5 and 2 ms^−2^, and 2 and 2.5 ms^−2^, a significant increase in drop-out was found between the survival curves of 1 and 1.5 ms^−2^ (Bonferroni corrected *p* = 0.0047), 1.5 and 2 ms^−2^ (*p* = 0.0107), but not between 2 and 2.5 ms^−2^ (*p* = 0.473). The hazard ratios were 1.64, 1.56, and 1.25, respectively, indicating a monotonic increase in the probability of dropout with increasing acceleration amplitude.

In this experiment, the most discriminative measure of how sickening a certain stimulus was given by the MISC rate. Figure 3B shows a monotonically increasing MISC rate on average across the group of participants (for the individual MISC rates of all participants, see Supplementary Figure S1). This monotonicity is further supported by the fact that a linear model provides a significantly better fit to the MISC rate data than a constant (intercept-only) model (AICc = –1.92 vs. AICc = 29.4).

**Figure 4:**
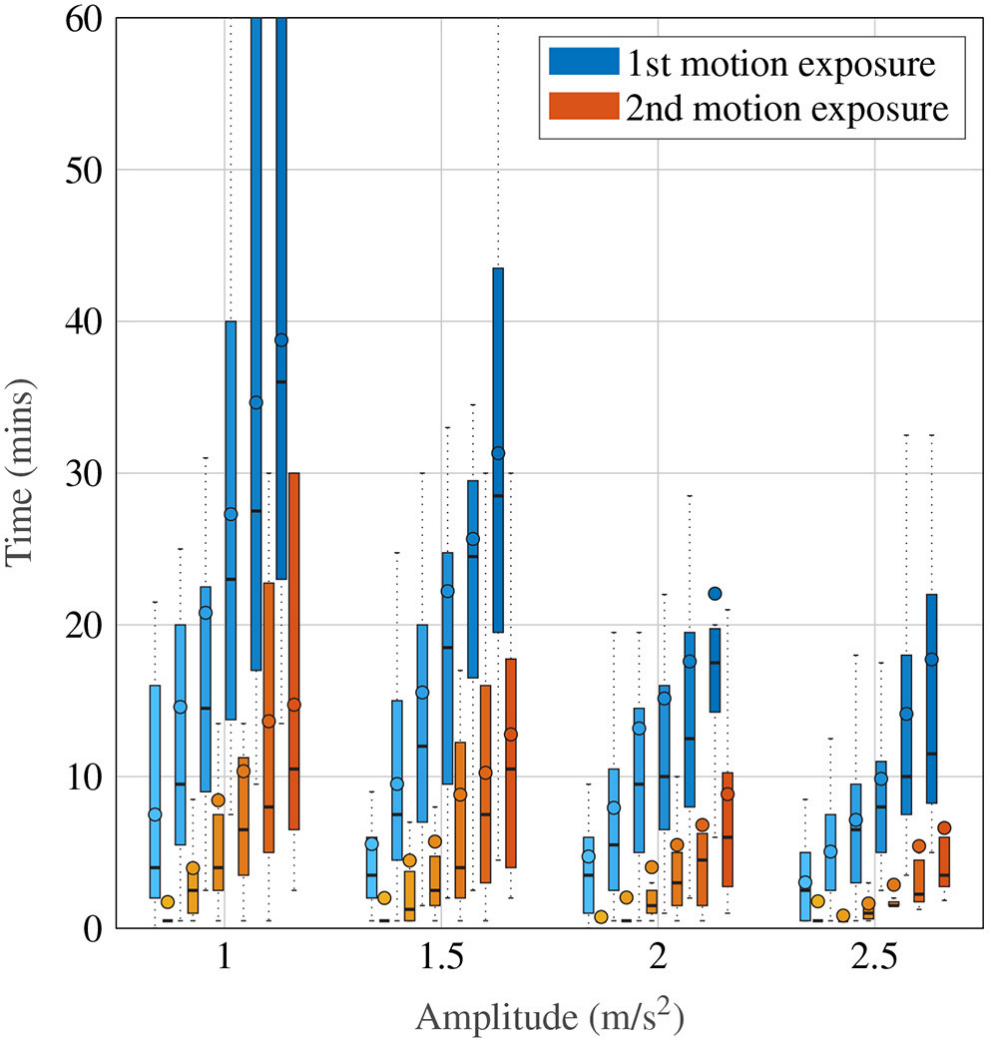
Time to reach a certain MISC level as a function of amplitude during the first and the second motion exposures, given by the blue and orange-shaded bars, respectively. The darker shades correspond to increasing MISC levels.

Figure 4 shows a more detailed breakdown of time to reach each a certain MISC rating, where the left-most lightest colored bar graph for each condition is for a MISC of 1 and the right-most darkest color is 6, for all tested amplitude conditions. Both data for the first (shade of blue) and the second motion exposure (shade of red) are presented. Figure 4 again shows that with increasing amplitude, there is a decrease in the time it took to reach a certain MISC level. Furthermore, the presence of motion sickness hypersensitivity is observed during the second motion exposure, shown in the yellow to orange colored bars, where time to a certain MISC rating is reduced by 61% on average compared to the first exposure.

### 3.2. Oman Model

As motivated in Section 2.5.2, three model variations were evaluated: the Session Fit Unit Power, Joint Fit Group-level power and Joint Fit Individual-level Power. The results for these cases are presented separately in this section.

#### 3.2.1. Session Fit, Unit Power

**Figure 5:**
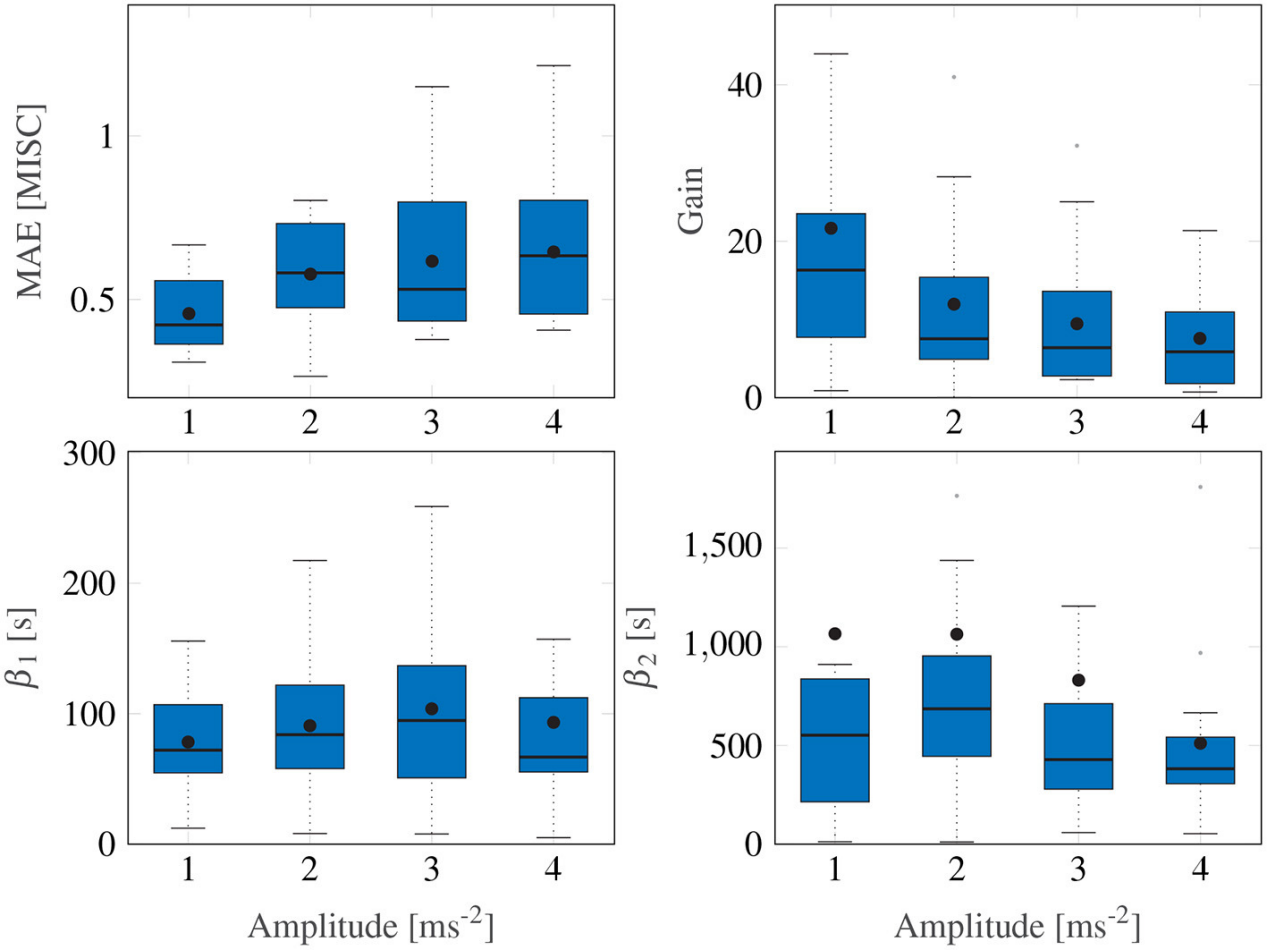
Box plots showing the mean absolute error, the gain and the two time constants of the Session Fit, Unit Power model variation. The Box plot is in standard form, with the center black line indicating the median, the dots indicating the means, and the sides of the box indicating the quartiles. Some outliers are above the maximum *y*-value limits chosen for the respective subplot.

For the Session Fit, Unit Power case, the Oman model is fitted to all amplitude conditions individually for each participant, as also done in Irmak et al. (2020). Figure 5 shows Box plots of the fitting errors (MAE), the gains, and the long and short time constants for each amplitude condition.

A Friedman test shows significant differences in the MAE, with an average of 0.54, (χ^2^ = 9.15, *df* = 3, *p* = 0.027) across motion amplitude conditions, meaning there is a significant difference between model fitting accuracy across the different amplitude conditions. A *post-hoc* test, however, shows no significant difference between any set of individual amplitude conditions. On average, *E*_*joint*_ is 0.94 (STD = 0.29).

Figure 5 shows a significant downward trend in the gain of the model with increasing amplitude (χ^2^ = 12.8, *df* = 3, *p* = 0.005). There were no significant differences in either the fast nor the slow path time constants across the amplitude conditions (χ^2^ = 4.05, *df* = 3, *p* = 0.26 and χ^2^ = 1.43, *df* = 3, *p* = 0.7, respectively). On average, β_1_ and β_2_ had median values of 73.6 and 510.4 s, respectively. The implication of this is that the fast and slow path time constants are seen to be acceleration amplitude invariant and can thus be considered a constant property of each individual.

#### 3.2.2. Joint Fit, Individual-Level Power

The Session Fit shows that the gains change as a function of input amplitude, whereas the time constants may be fixed. To get a single set of parameters (rather than amplitude dependent gains) that will predict across all amplitudes, the model requires an *output power-law* scaling. In the Joint Fit, Individual-level Power model variation, the dynamics of sickness with respect to input amplitude are given by allowing this output power scaling *p*_*o*_, to freely vary between individuals. This means that the amplitude sensitivity, just like both the gains and the time constants, is modeled as an idiosyncratic property unique to the individual.

For this model variation, the joint error *E*_*joint*_ was 1.01 (STD = 0.23), this is only marginally above the 0.94 of the Session Fit model variation (which is clear from the time domain plots shown in **Figure 7**), indicating that the model simplification from 12 to 4 parameters was successful.

#### 3.2.3. Joint Fit, Group-Level Power

In the previous model form, an individual power term was used. This power term can be fixed such that only three individual parameters are required to describe the motion sickness response, rather than four.

**Figure 6:**
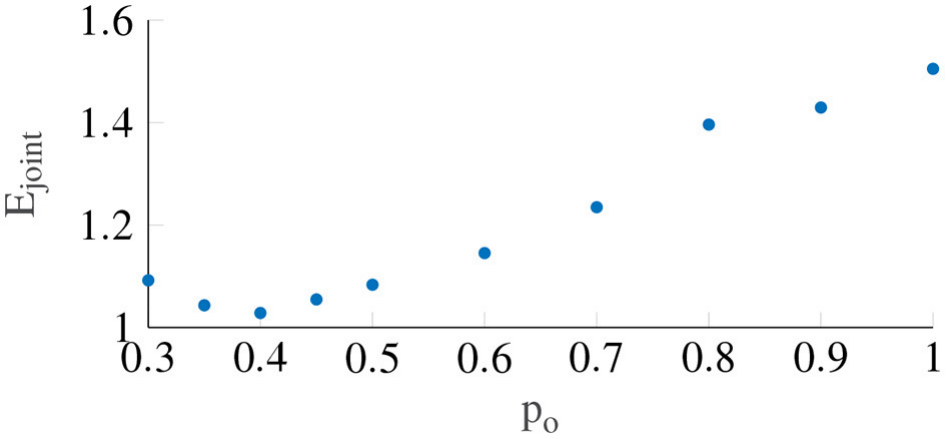
The error term *E*_*joint*_ with respect to the output power scaling, which is taken to be constant between participants. The lowest *E*_*joint*_ occurs when power is equal to 0.4.

Figure 6 shows the variation in the joint error term *E*_*joint*_ as a function of the output power scaling, which was fixed for the whole population. It can be seen that the error term is minimized to 1.028 (STD = 0.23) when the output power scaling *p*_*o*_ is 0.4. The medians of the other Oman model parameters for output power scaling were 66.2 and 502.4 s for the fast and the slow path time constants (β_1_ and β_2_), respectively, and 70.8 for the gain (*K*). Using the output scaling of 2 proposed by Oman (1982) led to an error of 2.54, higher than the minimum we find using an output scaling of 0.4.

Contrary to previous findings by Irmak et al. (2020), there was no correlation between the fast and slow time constants (r = 0.074). This may be explained by the fact that we tested multiple amplitudes rather than one in the current study, fitting all concurrently with an associated output-scaling term. This may have reduced any potential correlation between the two time constants. A second factor may be that the previous finding was a spurious correlation, which this study was not able to replicate. This is plausible because the two time constants in fact represent different classes of responses, hormonal and neural (Oman, 1982). These are likely to be independent and uncorrelated processes.

By setting *p*_*o*_ = 0.4 in equation 1, the relationship between the conflict magnitude and the predicted sickness output of the model is given by:


(3) $$\begin{array}{r} {\text{MISC}}_{ss}={\left(KA^{2}+A\right)}^{0.4} \end{array}$$

K is the Oman model gain and is usually large with a median value of 70.8. This means that the steady state sickness value predicted by the model has an approximately linear relationship to input motion amplitude ${\text{MISC}}_{ss}\approx K^{0.4}A^{0.8}$

#### 3.2.4. Fitting Comparison

The three variations of the model evaluated each have a joint error *E*_*joint*_ for each participant. These model errors (shown in Table 1) can be compared using the Friedman Test across models to evaluate whether their fit quality differs significantly. Doing so, the three tested models were found to differ significantly from each other (χ^2^ = 6.14, *df* = 3, *p* = 0.046) but this difference was marginal and indeed pairwise testing revealed no significant differences.

**Table 1:** Summary of fitting results, with the error averaged over participants *E*_*joint*_, the standard deviation over participants in the MAE, and the number of parameters for each model variation.

**Model variation**	**Average *E*_*joint*_**	**STD**	**# Parameters**
Session fitting, No power	0.94	0.29	12
Joint fitting, Individual-level power	1.01	0.23	4
Joint fitting, Group-level power	1.03	0.23	3

**Figure 7:**
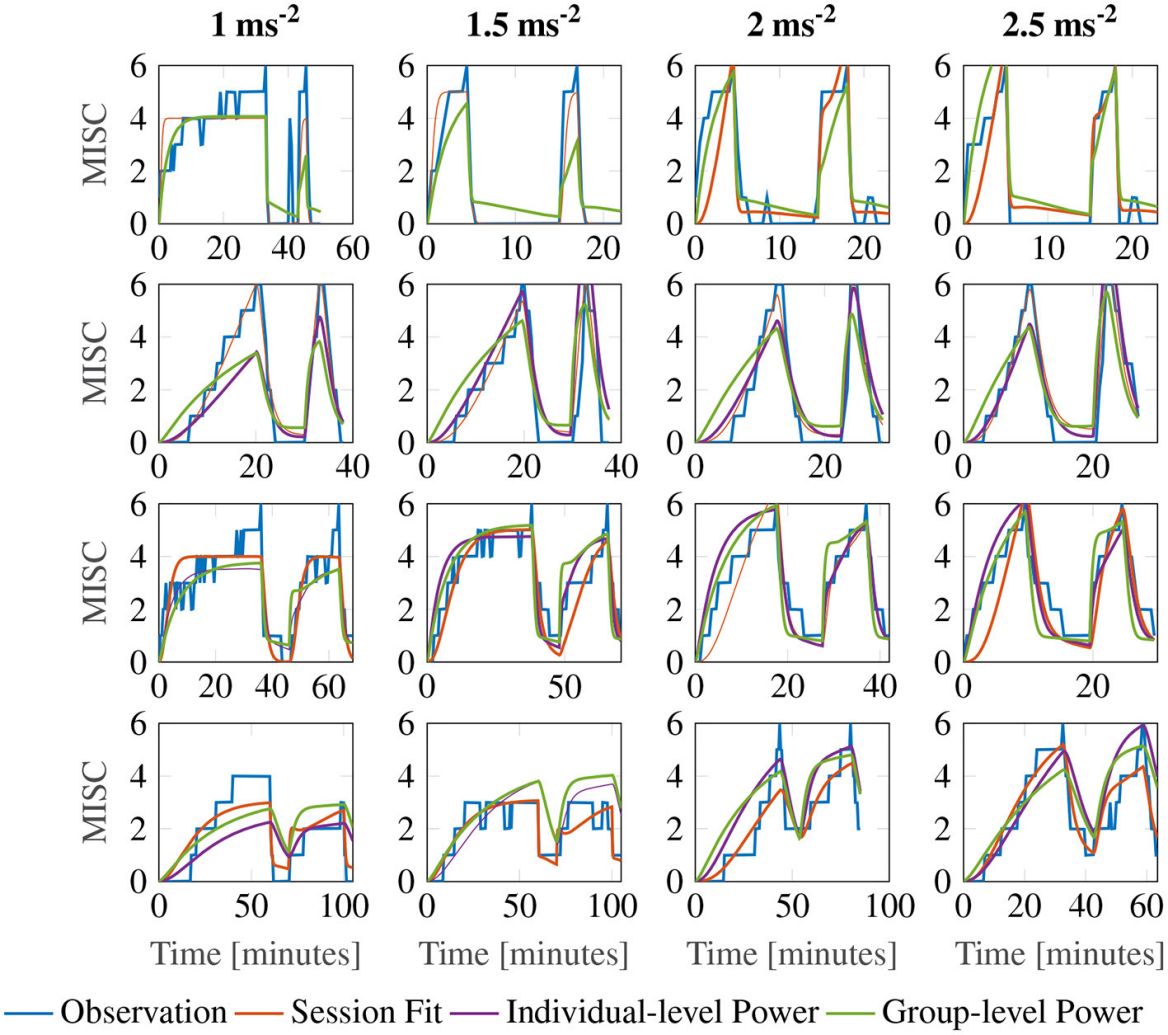
Representative sample of fittings for the three model variations for participants 11, 12, 13, and 14. The columns show responses for each amplitude condition, increasing in magnitude from left to right. The rows show results for each participant.

Figure 7 shows a representative sample of fittings for the three model variations for participants 11, 12, 13 and 14 (for the data of all participants, see Supplementary Figure S2). It is clear that there is little difference between the three model variations. It can therefore be concluded that individuals have time constants that are invariant of the motion amplitude, and that an output scaling of 0.4 allows the model to fit across multiple amplitude conditions just as well as fitting to a single session. This means that the 3 parameter model with the output power fixed across participants, but the gain and the time constants allowed to vary at the individual level offers a good compromise between fitting performance and model complexity.

### 3.3. Amplitude Cross Validation

Evaluation of model variations so far was with respect to how well they could fit the data. However, for a predictive model, it is also important to identify the capacity for generalizing to conditions not explicitly fitted to. We therefore performed cross-validation of the model between the different amplitude conditions. To do this, we looked at the mean MAE when we fitted to one, two and three conditions whilst predicting three, two and one condition. There were 4 combinations for the 1 fitting case, 6 combinations for the 2 fitting case and 4 combinations for the 3 fitting case, leading to 14 cross-validation data sets.

**Figure 8:**
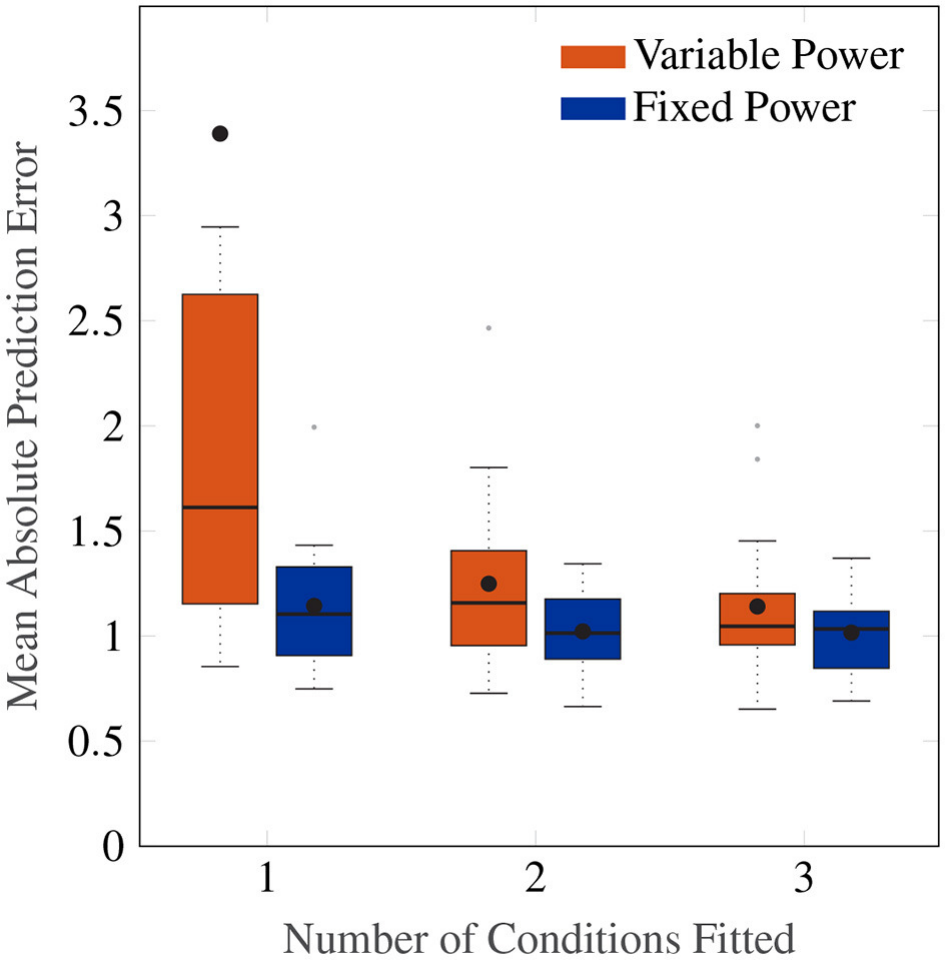
Box plot showing the mean absolute prediction errors for when 1, 2 and 3 cases are fitted to predict 3, 2 and 1 other conditions, respectively. The orange Box plots show prediction errors for the individual-level power law, and the blue Box plots show the prediction errors for the power law fixed to 0.4.

Figure 8 shows a Box plot of the mean absolute prediction errors for the procedure described above. Both models with an individual-level and a group-level power term variations show decreasing prediction errors with the number of conditions fitted. The group-level power model with *p*_*o*_ = 0.4 has overall a lower prediction error than the individual-level power model (Friedman test, χ^2^ = 12.3, *p* <0.001, *df* = 1), particularly for when fitting to data from only 1 amplitude condition.

Importantly, the group-level power on average has a fitting MAE of 0.90, which is close to the average prediction error of 1.15 after fitting only one condition. This indicates a high degree of regularity in the amplitude response that can be predicted by a power law of 0.4, and one that cannot be captured by the individual-level power model without larger amounts of data.

### 3.4. Sickness Forecasting

One of the properties of an effective predictive model is its ability to forecast future development of the modeled system's states. In this section, we evaluate this forecasting ability of the Oman model.

**Figure 9:**
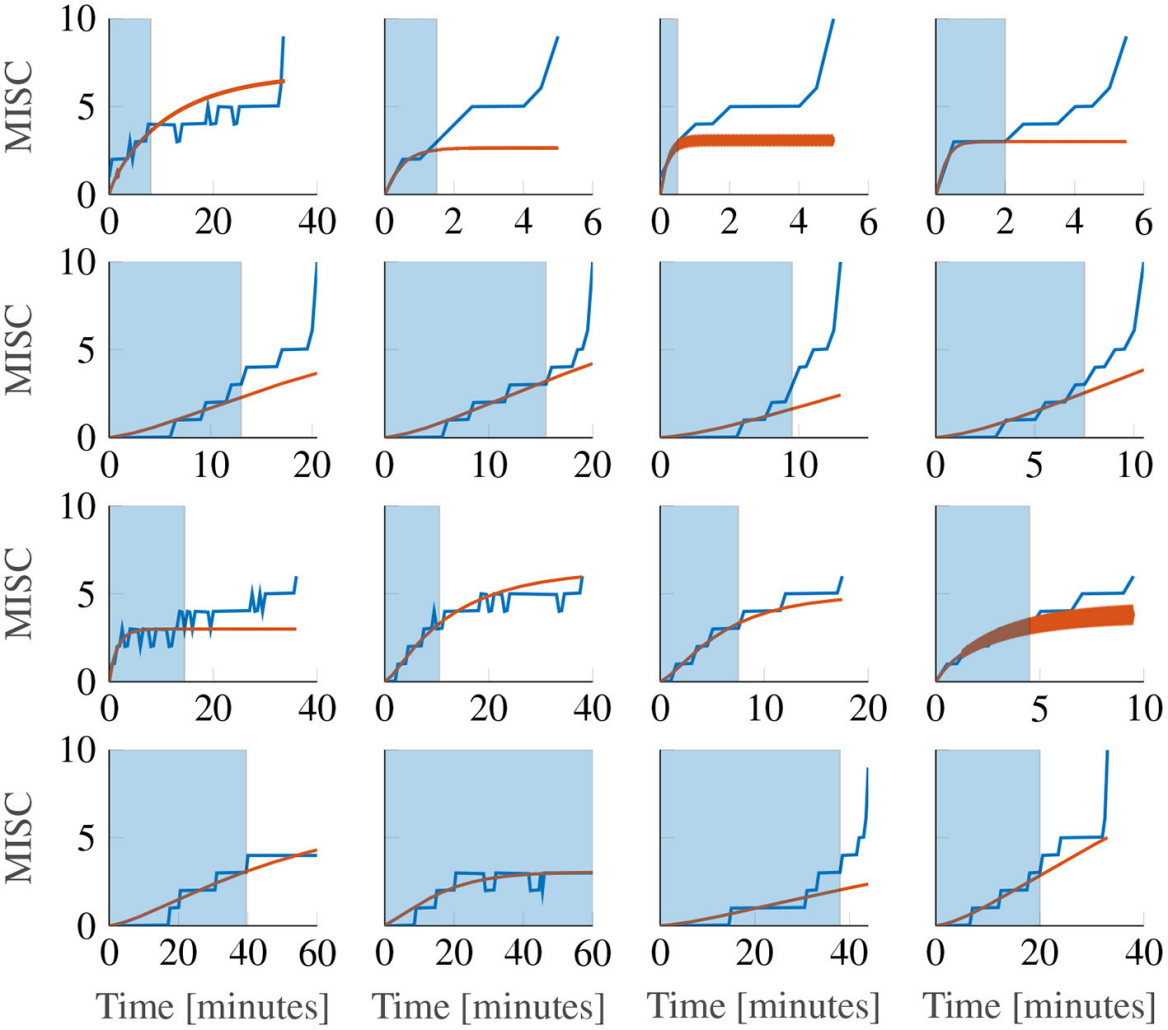
Representative sample of extrapolations from MISC 3 to the end of the first motion phase, for the Oman model. The columns show responses for each amplitude condition, increasing in magnitude from left to right. The rows show results for participants 11, 12, 13, and 14. The blue shaded area gives the observations the model uses to make forecasts.

Figure 9 shows the first motion phase responses of participants 11–14, where the rows represent the different participants and the columns the different amplitude conditions (for the data of all participants, see Supplementary Figure S3). In our experiment, participants 10, 11, 14 and 17 vomited or retched (MISC 10 and 9, respectively) very shortly after (<30 s) reaching a MISC level of 6. Because this occurred very shortly after reaching 6, in Sections 3.2 and 3.3, a MISC of 6 was taken as the end point of the experiment data used for fitting. In Figure 9 the full MISC trajectories are shown (blue lines), which for participants 11, 12, and 14 show a region of stable growth until a MISC of 6, then a blow-up to vomiting, as similarly reported in the results of Graybiel (1969).

This phenomenon cannot be captured by the Oman model, which, as noted before in Section 2.5, converges in a stable manner to a final sickness level that may be higher than 10. Predicting such high levels of sickness may not be a concern for most practical applications, for which the aim generally is to keep sickness at the lower MISC levels. Figure 9 shows Oman model predictions (in orange) when the model is fitted to all data up to a MISC value of 3 (blue shaded ranges) and sickness is then forecasted until the end of the experiment. It can be seen that this extrapolation from lower MISC levels in to the future for some participants suffers from premature convergence (e.g., participant 11 in Figure 9), where the model captures an initial seeming convergence of the MISC data to a final rating. This effect is explained by the small amount of data provided (only up to and including MISC 3) and the inherently convergent nature of the Oman model.

Overall, it is the diverging sickness trajectories that show the largest forecasting errors, e.g., participants 12 and 14 in Figure 9. This can be shown statistically, by fitting a model of the form


(4) $$\begin{array}{r} \text{MISC}=at^{b} \end{array}$$

as proposed in Irmak et al. (2020), where *t* is time since the start of exposure and *a*, *b* are model coefficients. The responses that can be described by *b*≥1 have a diverging sickness response with respect to time, whereas those with *b* <1 have a converging response. The fitted model had an average coefficient b of 1.085 (25-75th percentiles: 1.043–1.448). This means that the MISC is approximately linear with respect to time. When fitting the Oman model using a long fitting window, it can describe both converging and diverging responses equally well. This is despite its natural tendency to converge. This is because for divergent cases, the model estimates a very large steady-state value, meaning that the initial rising part of the response is able to approximate the divergent cases quite well. However, when forecasting from lower MISC levels, using a shorter observation window, it is seen that forecasting performance for divergent cases is significantly worse than forecasting for convergent cases. This can be seen by comparing a constant (intercept-only) and a linear mixed-effect model, relating the power term to the mean absolute error in the forecast region (AICc = 149.0 and 136.9, respectively). With a difference between AICc values >10 (Fab, 2014), it can be seen that the linear model is significantly better, meaning that the forecasting error increases with increasing divergence of the sickness response. The cause of the suboptimal forecasting is partly due to the model form, which is always stable, but also due to the limited data used for fitting and extrapolating from.

At the individual level, 15 out of 17 participants had MISC responses that were in at least 3 out of 4 conditions either consistently divergent or consistently convergent. The remaining 2 participants showed convergent MISC responses in half of the conditions and divergent responses in the other half. This means that, on average, individuals show a propensity toward one type of motion sickness trajectory. This supports the existence of idiosyncratic differences in the qualitative form of sickness dynamics. We did not find a difference in the power term between the motion conditions (χ^2^ = 1.2, *df* = 3, *p* = 0.75). This indicates that the divergent/convergent dynamics is not modulated by differences in the motion amplitude in the range explored in this study.

In our study, the MSSQ was correlated marginally significantly with overall sickness susceptibility (ρ = 0.50 *p* = 0.05). The overall sickness susceptibility was quantified by averaging the MISC rates during the first motion exposure of an individual for all amplitude conditions. This finding indicates the usability of the MSSQ for predicting sickness susceptibility and hence as a tool for participant selection. However, a better selection could be made by first perturbing the participants at 2.5 ms^−2^ until they reached a MISC of 3 which would on average take 8 min. The Oman model may then be used to estimate participants' susceptibility directly. Doing this on the data from the experiment, there is a very strong correlation between the Oman model estimation of susceptibility and the overall sickness susceptibility as computed from the average MISC rate for a participant (ρ = 0.72 *p* = 0.002). This level of predictability with respect to actual sickness susceptibility is directly useful in candidate participant screening. A better predictive model would have higher susceptibility discrimination, at even lower MISC levels, requiring less simulator time. Indeed, the correlation between the Oman model forecasting and overall susceptibility is not significant ρ = 0.15 (*p* = 0.58) when data to only MISC of 2 is considered.

## 4. Discussion

This study investigated the amplitude and temporal dynamics of motion sickness at both the group and the individual levels. Participants underwent fore-aft sickening motions at four different acceleration amplitudes. Motion sickness development over time was reported using the MISC scale. First, using the dropout percentage and the MISC rate, the group-level response to varying amplitudes was evaluated. Also, three variations of the Oman model of nausea were used to characterize the dynamics of motion sickness at the individual-level. This was done by both fitting observed sickness at different amplitudes, but also by assessing the cross-amplitude validity of the model. Lastly, we investigated how well the Oman model can forecast future sickness based on a shortened measurement of initial sickness development.

### 4.1. Group-Level Observations

For the group-level response to increasing acceleration amplitudes, *we found a significant increase in sickness severity with increasing acceleration amplitude*, and hence sensory conflict magnitude, on the development of motion sickness. As seen in Figure 3, not only was this effect monotonous with respect to the acceleration amplitude, it could also be accurately characterized by a linear relationship, which was shown by comparing a constant mixed-effect model of MISC rate with a linear mixed-effect model (AICc = –1.92 vs. AICc = 29.4).

Previous studies by Griffin and Mills (2002), O'Hanlon and McCauley (1973), and Alexander et al. (1947) also reported a monotonic increase in sickness with respect to acceleration amplitude. In the study of Griffin and Mills (2002) only low-amplitude motions in the range of 0.4–1.56 ms^−2^ were used, and only the last two conditions were significantly higher in sickness severity from the baseline case of no motion. Therefore, a functional relationship between motion amplitude and sickness could not be formulated. The studies by O'Hanlon and McCauley (1973) and Alexander et al. (1947) assessed vomiting incidence at the end of their experiments (MSI) and found a log-normal relationship between MSI and acceleration amplitude. However, it would be incorrect to say sickness itself exhibits log-normal behavior for the range of accelerations used in these studies. Indeed, in our study, we report both the dropout rate and the rate of sickness development. For the dropout rate, which is a similar metric to the MSI, dropout percentages for 2.5 and 2 ms^−2^ are not significantly different, whereas MISC rate indicates a linear, rather than a log-normal, relationship between sickness and acceleration amplitude. The data of Lawther and Griffin (1988) suggest that this linear relationship may continue down to the range of 0.1–0.7 ms^−2^, i.e., to lower amplitudes than tested in our experiment. At the lowest acceleration magnitudes, i.e., below 0.1 ms^−2^, experienced sickness did not differ from the stationary case. This apparent ‘sickness threshold' is equivalent to reported translational acceleration perception thresholds (Gianna et al., 1996; Heerspink et al., 2005). As remarked previously, acceleration is often used as a proxy for sensory conflict for experiments lacking visual stimuli, and in our current experiment set up the two are assumed to be proportional to each other. Very sickening stimuli, such as the cross-coupled coriolis, which can elicit vomiting in minutes as opposed to >10 min as in this study, likely produce much higher sensory conflicts, which may be translated to an equivalent acceleration, indicating that the monotonic amplitude relationship likely holds at even higher accelerations than 2.5 ms^−2^. Approximately 95% of all vehicle accelerations are within the maximum acceleration used in this study (Feng et al., 2017). Therefore, we can conclude that linearity in the sickness response can be an adequate modeling assumption at the group-level for automated vehicles.

With respect to the metrics used to quantify group-level responses, we chose the drop-out rate and MISC rate. The drop-out rate provides an easy to interpret measure of sickness, whilst also allowing us to perform survival analysis. For our experiment, the MISC rate is less directly dependent on our selected termination criteria. It is defined as the MISC rating at the end of motion exposure, divided by the time in minutes to this final value. In the current study, we fitted the model *at*^*b*^ to all MISC responses; the resulting average value for *b* is 1.085 (25-75th percentiles: 1.043–1.448). This means that the MISC is approximately linear with respect to time, increasing monotonously with respect to time, with no long-duration decreases. Therefore, computing the average gradient of the MISC curve, i.e., our MISC rate, is an appropriate parametrisation of the response.

In cases such as naturalistic driving where such monotony is not observed, a model-based approach may be more appropriate. The kind of model used for this purpose is a formal accumulation model, such as the Oman model. This is because in such a scenario, the sickness response will be complex and time-varying, depending on the accelerations encountered. Using traditional ways of parametrising the sickness response will make both within- and between-participant comparison difficult, requiring, in the least, many sessions to average across a representative sample of acceleration exposures. With a model-based approach, the parameters of the fitted model will be invariant with respect to the motions encountered and easier to compare.

### 4.2. Individual-Level Modeling

In this study, we showed that motion sickness development over time could be accurately modeled at the individual level, for the different tested amplitude conditions separately, with a modified version of Oman's sickness model. We found that the time constants of sickness development were approximately motion amplitude independent, with median time constants of 66.2 s and 502.4 s for the model's ‘fast' and ‘slow' time constants, respectively. One concern of automotive engineers in utilizing the findings of motion sickness studies could be the fact that usually the motions encountered in these studies are aggressive, with the intent of quickly making participants motion sick, whereas motions that lead to motion sickness in vehicles tend to be more gradual and accumulate over the span of up to an hour. In this study, we tested both aggressive motions (2.5 ms^−2^) and gentle motions (1 ms^−2^). The fact that no difference in the time constants was found implies that the temporal dynamics of motion sickness are amplitude-independent, with only varying scaling factors affecting the final level of sickness. This suggests that the findings of sickness studies, all else equal, can be directly applied to automotive control and design.

In this study, we report only on nonlinear output scaling in Oman's model. However, we also investigated the use of output scaling, see Figure 2. The original Oman model has the conflict vector as an input, which is processed by the two paths, whose outputs are then summed. This summed output is the *latent* sickness. The output power scaling transforms this latent sickness in to a subjective magnitude estimate, via an exponential term corresponding to Steven's power law (Stevens, 1946), which maps stimulus intensity to perceived intensity. As employed by Bos and Bles (1998), the reasoning for input scaling is different. Here, the conflict signal itself is assumed to be remapped with a nonlinear scaling (sigmoid), where small sensory conflicts remap to zero and large conflicts are saturated. We approximate this through an input power law. We found that the output power law provided a much better fit to our data, with a mean joint error (*E*_*joint*_) of 1.03 compared to 1.4 for the input scaling. As Figure 6 shows that with output scaling for all powers up to 0.8 the joint fitting error is below this optimum result for input scaling, we conclude that output scaling on the modeled latent sickness metric is superior for our experiment data.

**Figure 10:**
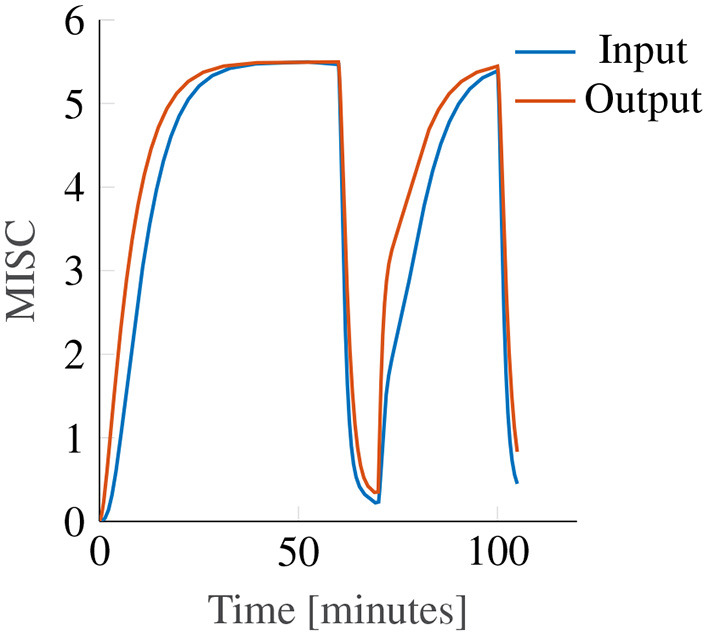
Example showing the effect of input (blue line) and output (orange line) power scaling on the predictions of the Oman model for constant amplitude input.

While both with input and with output scaling Oman's model can model convergence to an identical final steady-state sickness level (with adapted gain and power law exponent), see Figure 10, this will always result in differences in the temporal dynamics. In these example model responses, the input and output power scaling, as well as the short and long time constants, are all the same, while the model gain is adjusted such that both modeled responses converge to the same steady-state sickness. Regardless, it can be seen that for constant amplitude stimuli, the output scaling responds faster to the input, particularly in the hypersensitivity phase. This is because the output of the slow and fast paths accumulate more slowly when the input signal strength is reduced by an input scaling, than when it is not. The reason why this steeper increase may better represent our data is that the MISC is on an ordinal scale. Participants are likely to spend less time at the lower ends of the MISC scale, which represent smaller increments on a scale of subjective discomfort (de Winkel et al., 2022), than the higher end of the MISC scale. This is particularly relevant for the hypersensitivity phase.

As shown in Figure 6, an output scaling of 0.4 provided the best fit to our dataset. The power of 0.4 approximately linearizes the conflict to sickness relationship, where for large values of *K*, Equation (3) reduces to MISC${}_{ss}\approx K^{0.4}A^{0.8}$. Likewise, for the input scaling this optimal power was found to be similar, i.e., 0.5. This value fully linearizes the conflict to sickness relationship. This means that irrespective of the scale used to measure sickness the Oman model, or any other model of sickness accumulation, should have a power-law relationship that enforces linearity, whereby only the gain of the system is expected to change depending on the rating scale used. Finding the system gain for the different rating scales would greatly contribute to comparing and generalizing the findings of different motion sickness experiments.

For the first time, this study evaluated forecasting/predicting sickness at the level of the individual, based on a short duration of initial sickness data (MISC <3). The development of sickness over time in the first motion exposure could be predicted accurately, with a MAE of 0.93. In our previous work (Irmak et al., 2020), we identified two groups of participants by fitting a power law to measured MISC as a function of time (see equation 4). The participants for whom the exponent *b*≥1 were classified as *divergent* and those with *b* <1 were classified as *convergent*. In that study, the Oman model was able to fit both groups equally well, which cast doubt on whether these two groups were indeed qualitatively different. However, in the present study we found that the Oman model had significantly higher accuracy when *predicting* convergent, as compared to divergent cases, thus supporting the notion that MISC trajectories are indeed qualitatively different between individuals.

One notable property of the Oman model that affects forecasting of future motion sickness is that it is always stably convergent. That is, there is a steady-state sickness value *MISC*_*ss*_ that it will converge to as time tends to infinity. This means that if the participant has converging dynamics, and sickness is observed until a MISC of 3, the model will predict a convergence to approximately a MISC of 3. However, it is known from the data that this is not the case, and that participants likely continue to become more sick, especially if they have reached moderate sickness relatively quickly. A striking example of this issue is shown in Figure 9 for the first participant. Here, the observations indicate convergent dynamics, even though the participant reaches a MISC of 4 in under 4 min. This participant will inevitably vomit in finite time. The model, however, cannot account for this. Moreover, some participants (such as the first participant of Figure 9) show convergent sickness behavior at first, followed by a sudden increase toward vomiting. There can be multiple reasons for this. One explanation is that the participants use the MISC scale as a subjective discomfort scale, and that for these participants, a MISC of 6, which was the termination criterion in this study, was seen as the point after which they could not continue because they would otherwise vomit. Alternatively, it can also be that these participants experienced an unstable “avalanche” of symptoms. Such an effect has been reported in literature (Graybiel, 1969; Bock and Oman, 1982). To account for both the stable and unstable behavior seen in motion sickness responses, the next step should be to model the dynamics of sickness as a *bistable* system. In this, participants may have two equilibrium points. One that is at a level of sickness below 10, depending on motion amplitude, and the other at a MISC level of 10. Modeling this requires higher degree non-linear differential equations than included in Oman's model.

Sickness forecasting as demonstrated in this paper is not only a theoretical exercise, but has methodological implications for both motion sickness studies and applications such as the individualization of automated driving algorithms. In this study, we showed that using the Oman model and a short duration perturbation at the highest amplitude, one can reliably predict the overall motion sickness susceptibility of an individual. Such a paradigm can be used as a screening method for ensuring participants of similar sickness susceptibilities are enrolled in to motion sickness studies. This would for instance increase the power of studies comparing different mitigation methods. Moreover, such a method provides a basis for individualized and online sickness-mitigating adaptive tuning of automated driving algorithms.

Well-known models of motion sickness development contain two components, being 1) Conflict models predicting conflicts resulting from sensory integration, and 2) Accumulation models predicting motion sickness development in time. One example of a motion sickness model is the subjective vertical conflict (SVC) model (Bos and Bles, 1998; Wada, 2021). The conflict generation part of the model is based on the difference between the sensed vertical and the subjective vertical, which is thought to drive motion sickness. It is a specific implementation of the idea of sensory-expectancy conflict put forth in Oman (1982). Conflict models are needed to capture sensitivity toward complex motion stimuli, including multiple motion directions and frequencies. The conflict derived in the SVC is then accumulated using a simple second order filter. This accumulation model is less sophisticated than the Oman model used in this study, which is able to describe hypersensitivity.

Our study focuses on motion sickness accumulation in time for a single degree-of-freedom motion stimulus, i.e., a 0.3 Hz sinusoidal fore-aft seat motion. For such a simple stimulus, conflict models degrade to a simple gain, where the exact gain (proportion of fore-aft acceleration that is attributed to the subjective vertical) is dependent on the stimulus frequency and the subjective vertical time constant.

In our approach, this gain is (implicitly) identified in our fitting of Oman's model. Therefore, if the actual sensory conflict would be only 50% of the input motion, then the gain of our fitted model would simply be 50% larger. Thus, our method of using acceleration as the input to Oman's model is, for our specific stimulus, equivalent to the conflict between the sensed and the expected vertical that can be derived from the SVC model.

One important application of this work is that now that the relationship between conflict and the subsequent sickness is known, the system that maps motion inputs to sensory-conflict can be identified by using closed-loop system identification techniques (Rojas et al., 2007; Qian et al., 2016).

### 4.3. Limitations

As discussed previously, particularly at the lower amplitudes, there might be an amplitude threshold below which people do not experience motion sickness. In this study, the range of the amplitudes studied was between 1 and 2.5 ms^−2^. In future studies, it is essential to also include lower amplitudes to also gain an improved quantitative understanding of motion sickness severity and temporal dynamics for low-amplitude stimuli. In addition to increasing the range of accelerations for which our motion sickness models are effective, experiments that include a high number of different motion amplitudes measured within the same experimental session, as representative of real vehicular transportation, would further help to strengthen motion sickness model validation.

In the present study, the model successfully described hypersensitivity after a 10-min break. In a previous study, Irmak et al. (2020) the break duration was until the participant reached a MISC of 2, which only very rarely exceeded 10 min. This means that the model can describe hypersensitivity observed after break durations up to 10 min. One limitation is therefore the lack of data to verify whether the same modeling accuracy is retained for longer rest durations. Being able to model these longer rest durations may not be relevant for short distance journeys, however, it may be useful for predicting motion sickness during multi-stage long distance travel.

Lastly, it is likely that the amplitude and temporal dynamics found in this study do not depend on the direction of motion. Therefore, pure vertical and lateral motions will likely have similar time constants, gains and output power. This is given by the fact that the severity of sickness in different directions is similar to each other (Donohew and Griffin, 2004). However, if a coupling exists between different degrees-of-freedom, such that the resultant stimulus has a complex frequency spectrum, this may cause currently unknown interactions in the conflict signal due to differing frequency sensitivities (Irmak et al., 2021). Similarly, with reduced motion predictability (Kuiper et al., 2020) compared to our current sinusoidal acceleration stimuli, a greater sickness response is expected. In these cases, particularly the gains of the accumulation model may need to be calibrated.

## 5. Conclusion

This study investigated the individual amplitude sensitivity in motion sickness caused by sensory conflicts induced by fore-aft accelerations. At the group-level, we found that sickness severity increases linearly with acceleration amplitude between 1 and 2.5 ms^−2^ and argue that it does so for all relevant acceleration amplitudes in vehicular transport. From fitting a modified version of Oman's model of sickness progression, we found that, at the individual-level, sickness on average increased linearly with acceleration amplitude, even though some participants exhibit higher or lower order amplitude sensitivities. Importantly, we note that the time constants governing motion sickness development vary between individuals, but are independent of the acceleration amplitude. Furthermore, our data shows that a group-level fixed output scaling with an exponent of 0.4 enables Oman's model to inherently account for stimulus amplitude variations, as considered in our tested amplitude conditions. Lastly, we showed that the Oman model can be used to forecast the temporal evolution of sickness beyond a brief observed initial exposure. In this we found that forecasting works better for convergent, rather than divergent responses, this is largely due to the inherently convergent dynamics of the model. Overall, these findings enable improved modeling of motion sickness accumulation in mixed acceleration environments, such as traffic, and better participant prescreening for motion sickness experiments, as well as tuning of automated driving algorithms for individual passengers.

## Data Availability Statement

The original contributions presented in the study are publicly available. This data can be found here: https://doi.org/10.4121/18133973.v1.

## Ethics Statement

The studies involving human participants were reviewed and approved by Human Research Committee, TU Delft under application number 1425. The patients/participants provided their written informed consent to participate in this study. Written informed consent was obtained from the individual(s) for the publication of any potentially identifiable images or data included in this article.

## Author Contributions

TI contributed to conception and design of the study, contributed to data analysis and modeling work, performed the experiments, and wrote the manuscript. VK contributed to conception and design of the study, contributed to initial data analysis and modeling work, and performed the experiments. RH contributed to conception and design of the study, contributed to data analysis, and modeling work. KW contributed to conception and design of the study, contributed to data analysis, and modeling work. DP contributed to conception and design of the study, contributed to data analysis and modeling work, and facilitated the simulator experiment's implementation. All authors contributed to manuscript revision, read, and approved the submitted version.

## Funding

This work was funded by I-AT Interreg Automated Transport.

## Conflict of Interest

The authors declare that the research was conducted in the absence of any commercial or financial relationships that could be construed as a potential conflict of interest.

## Publisher's Note

All claims expressed in this article are solely those of the authors and do not necessarily represent those of their affiliated organizations, or those of the publisher, the editors and the reviewers. Any product that may be evaluated in this article, or claim that may be made by its manufacturer, is not guaranteed or endorsed by the publisher.
