# Supplementary material for: Amplitude and Temporal Dynamics of Motion Sickness
Source: Front Syst Neurosci. 2022 May 9;16:866503. doi: 10.3389/fnsys.2022.866503 (PMC9126086; doi:10.3389/fnsys.2022.866503)
Supplement: Supplementary file 1 [file Data_Sheet_1.PDF]

Supplementary Material

1 SUPPLEMENTARY FIGURES

1.1 Individual Normalised MISC Rate

Figure S1 shows the normalised MISC rate in the first motion exposure as a scatter plot with respect to the amplitude condition for all individuals that took part in the study. The MISC rate is normalised against the maximum MISC rate observed for a participant across all of their conditions. This gives a better representation of the amplitude sensitivity of the participants.

1.2 Model Fits to All Individual Responses

Figure S2 shows the fits of the three model variations for all participants and all conditions.

2 SICKNESS FORECASTING

Figure S3 shows extrapolations from MISC 3 to the end of the first motion phase, for the Oman model (in orange) and real observation (in blue) for all participants.

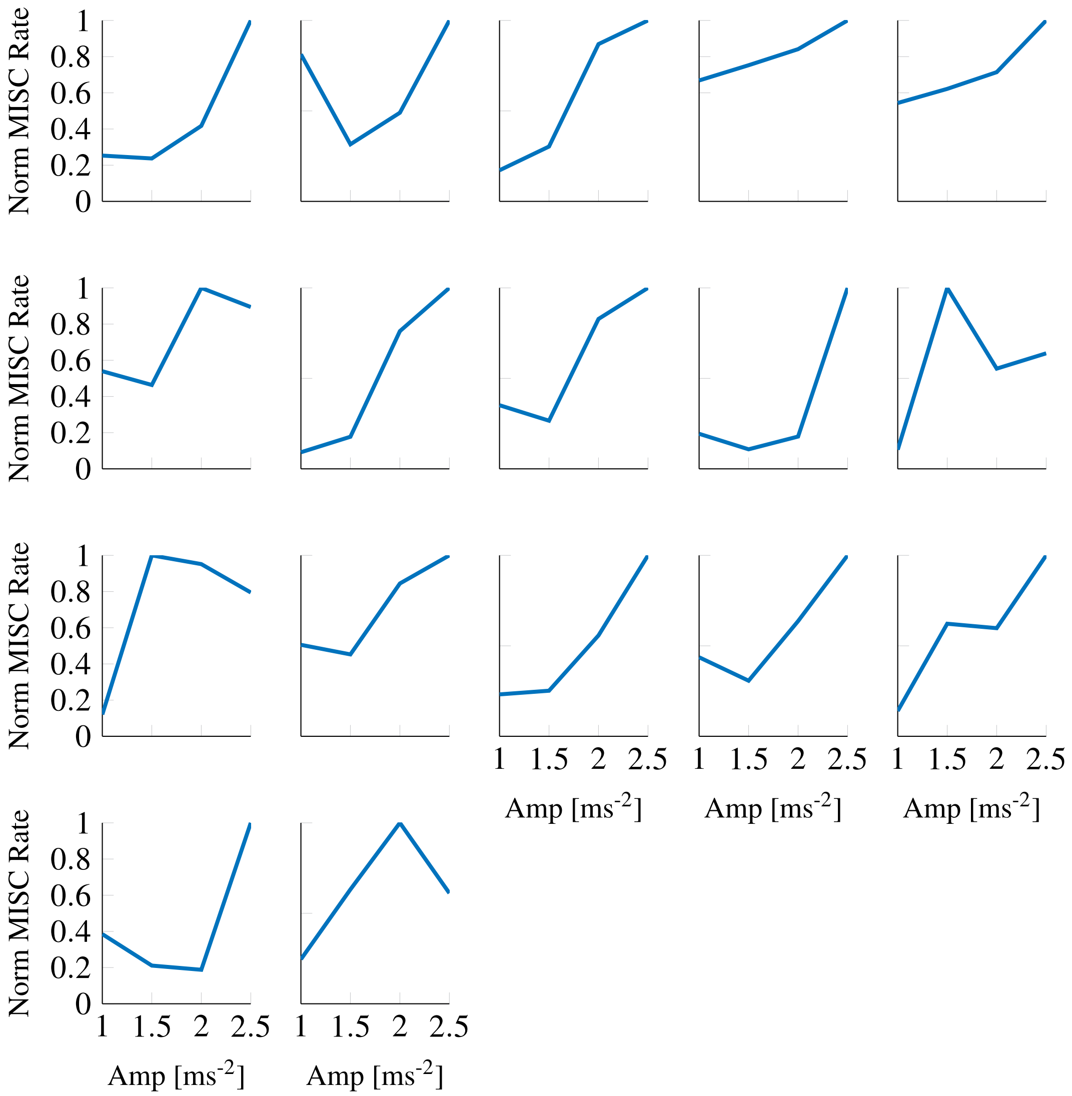

Figure S1: Normalised MISC rate obtained from dividing the MISC at the end of the first motion exposure with the time to the end of the first motion exposure, shown for all individuals

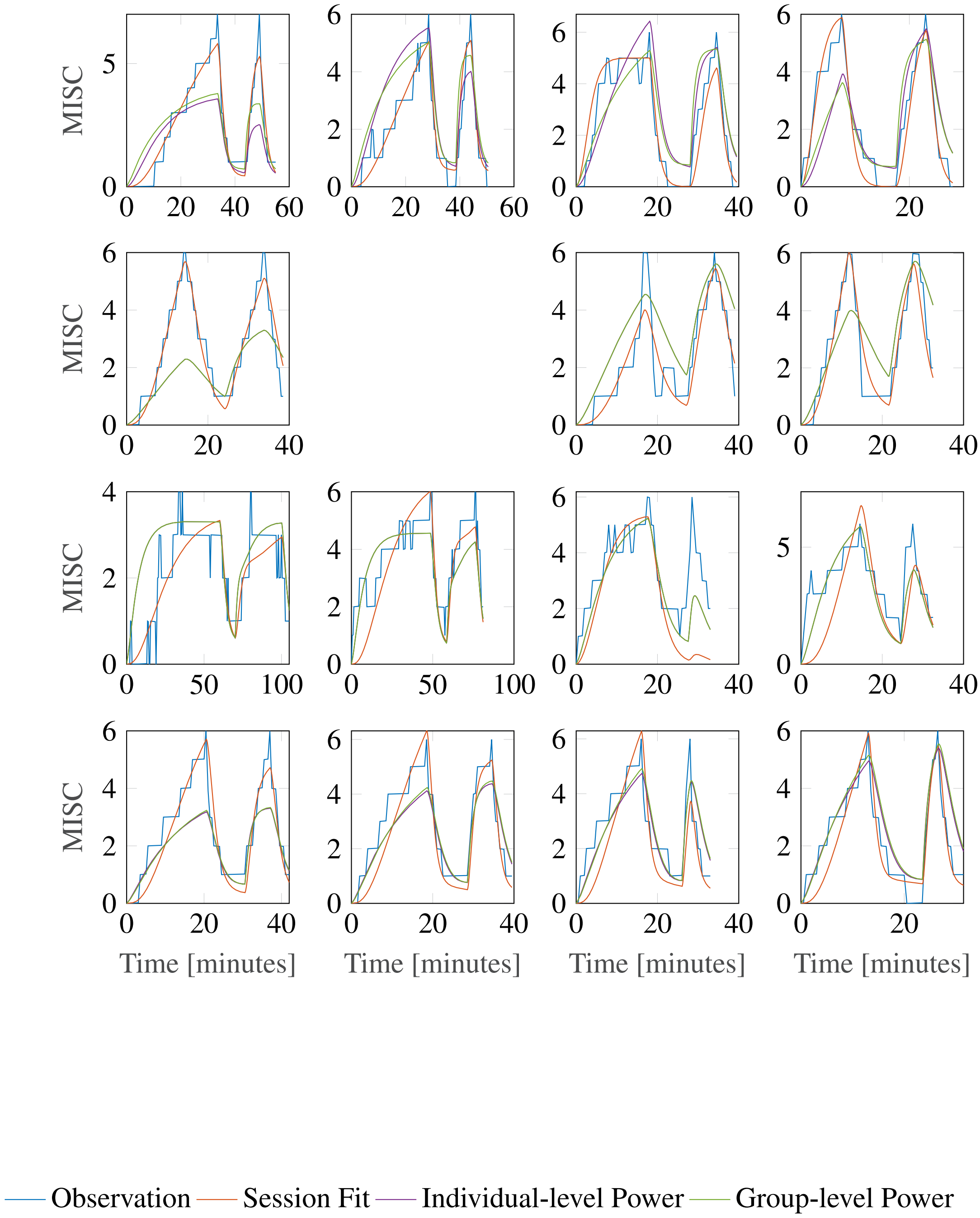
MISC
Time [minutes]
Observation
Session Fit
Individual-level Power
Group-level Power

(a)

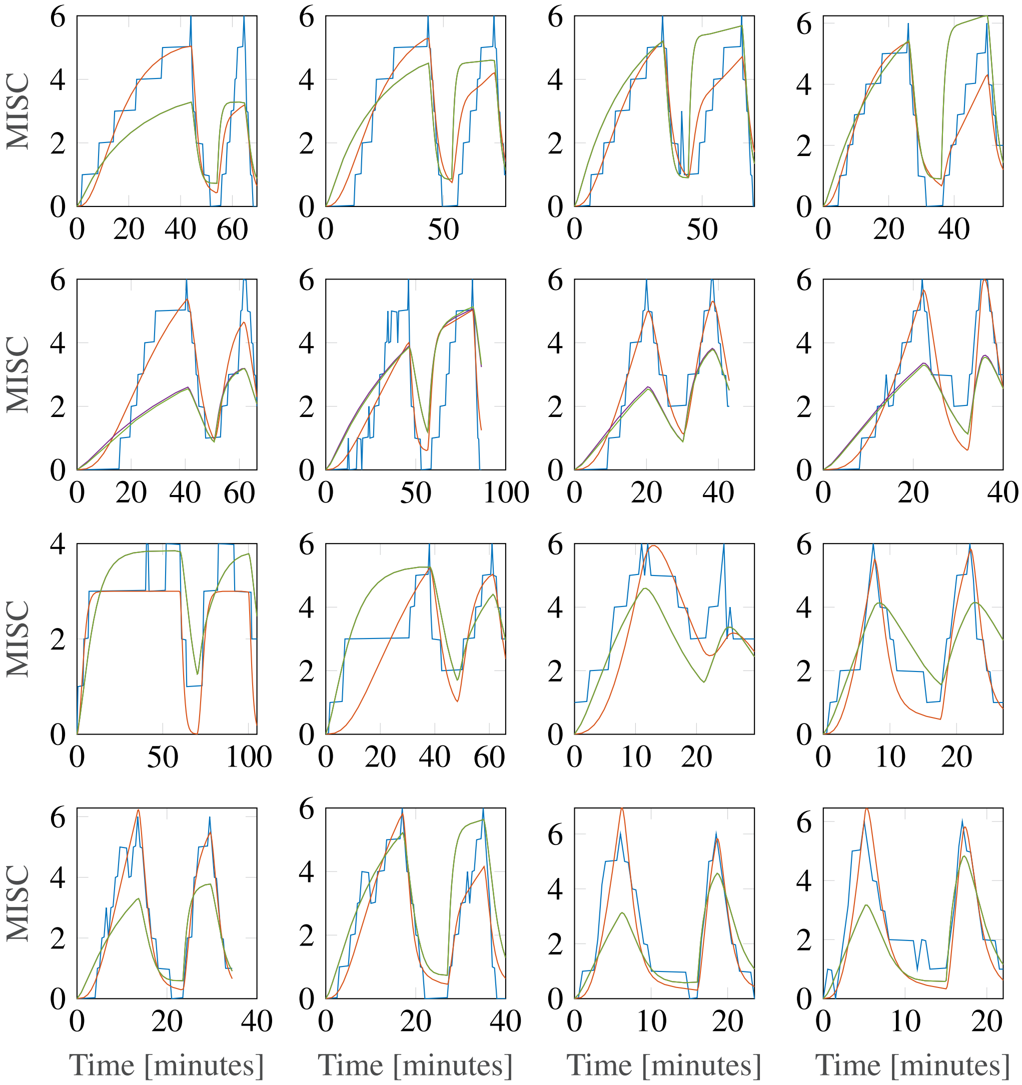
MISC
MISC
MISC
MISC
Time [minutes]
Time [minutes]
Time [minutes]
Time [minutes]

(b)

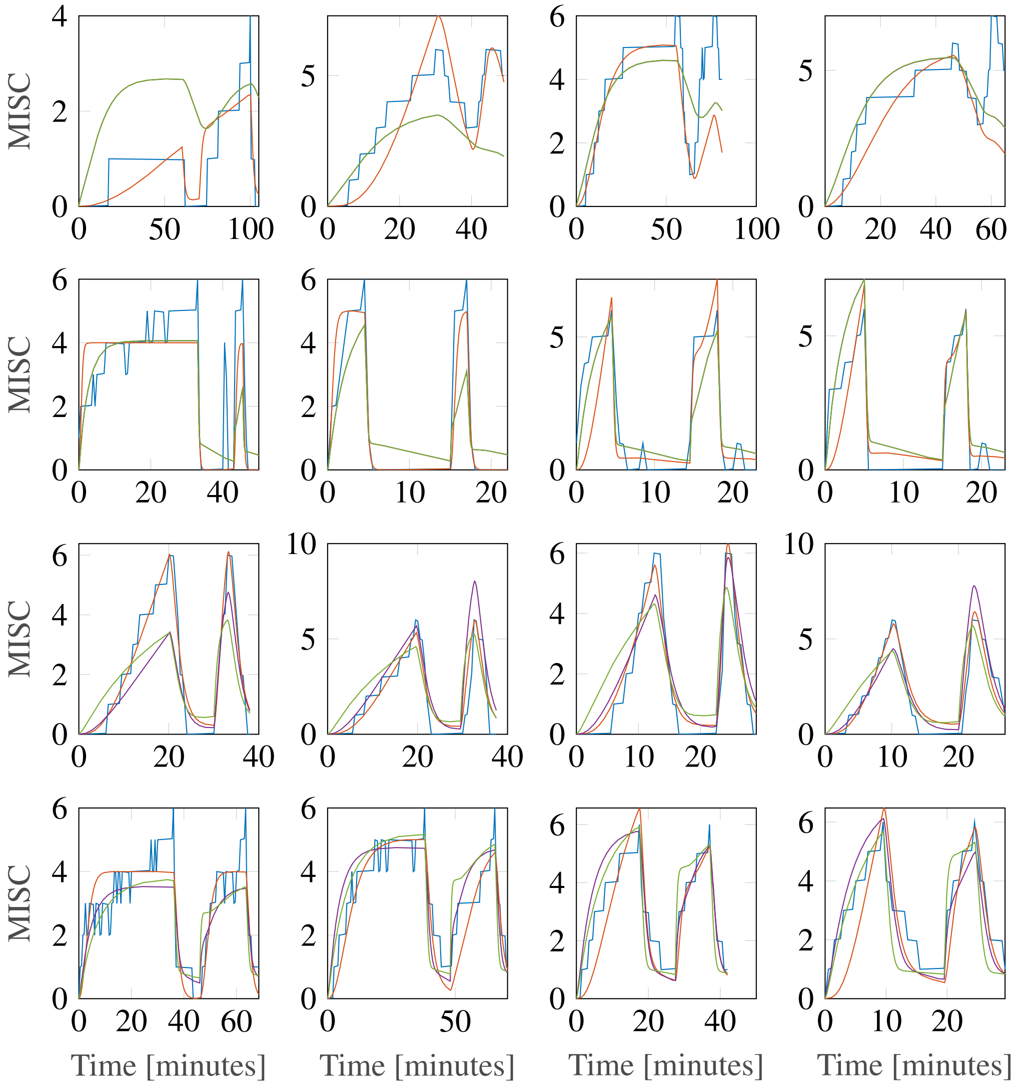
MISC
MISC
MISC
MISC
Time [minutes]
Time [minutes]
Time [minutes]
Time [minutes]

(c)

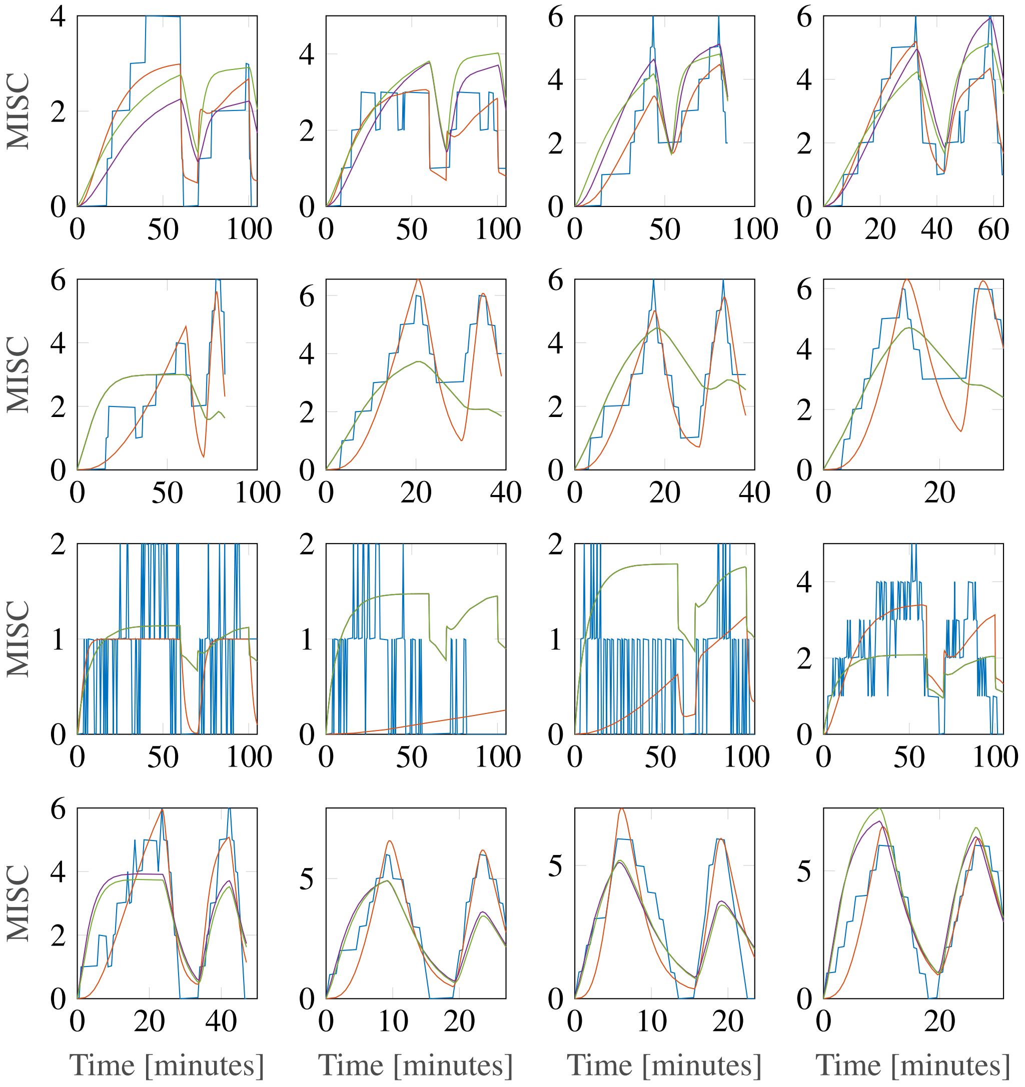

(d)

Figure S2: Three variations of the Oman model fitted to all sessions of all individuals. Each row is an individual participant, and each column is a motion condition, with increasing amplitude going from left to right

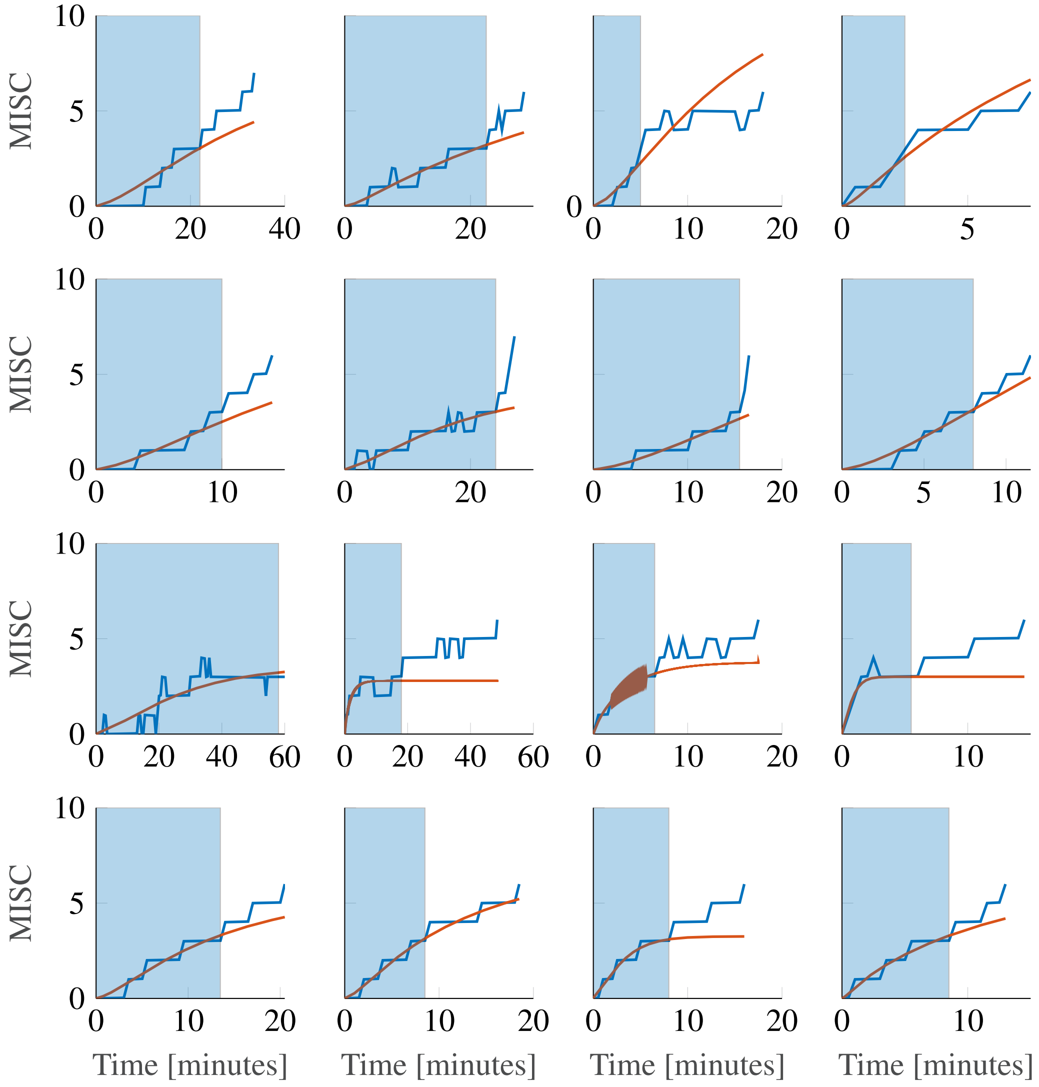
MISC
MISC
MISC
MISC
Time [minutes]
Time [minutes]
Time [minutes]
Time [minutes]

(a)

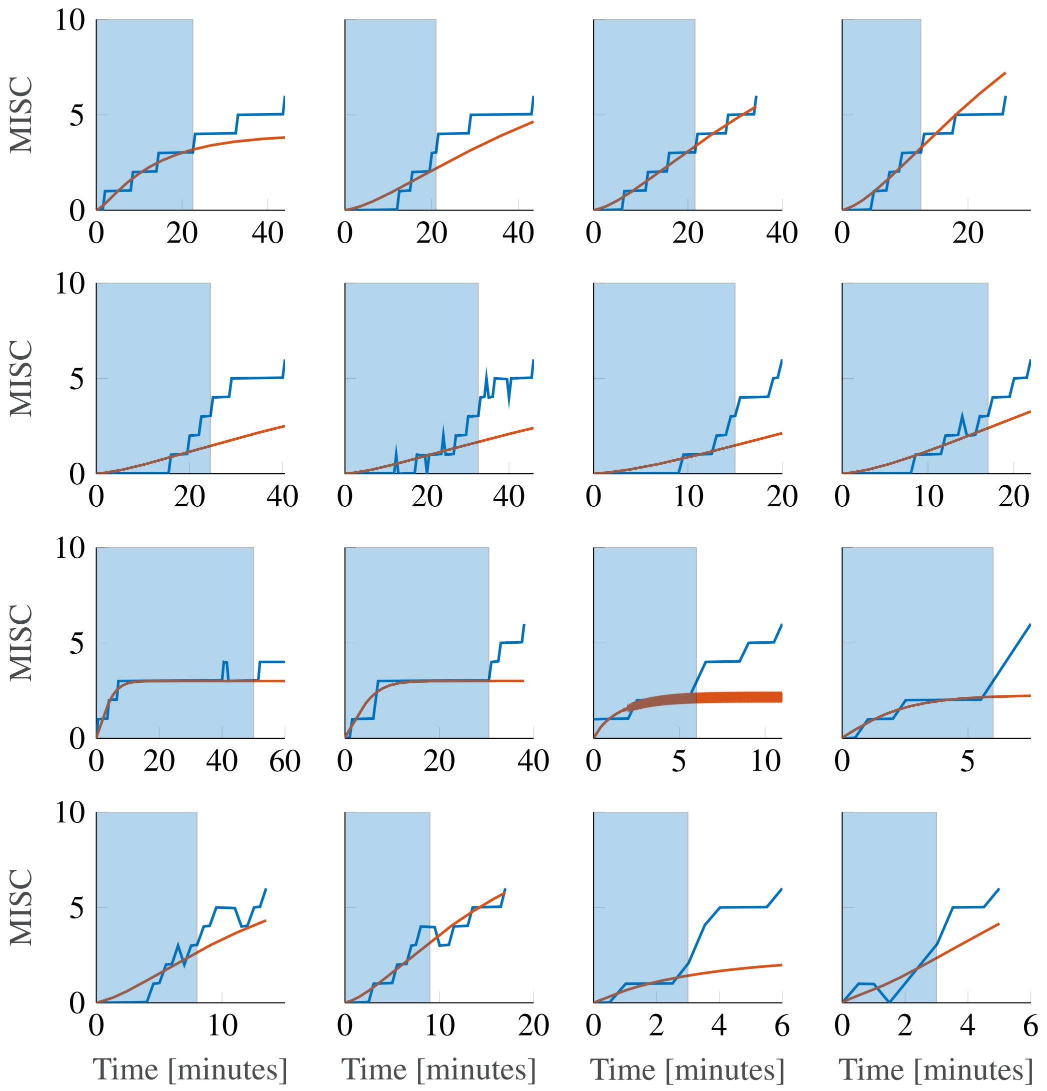

(b)

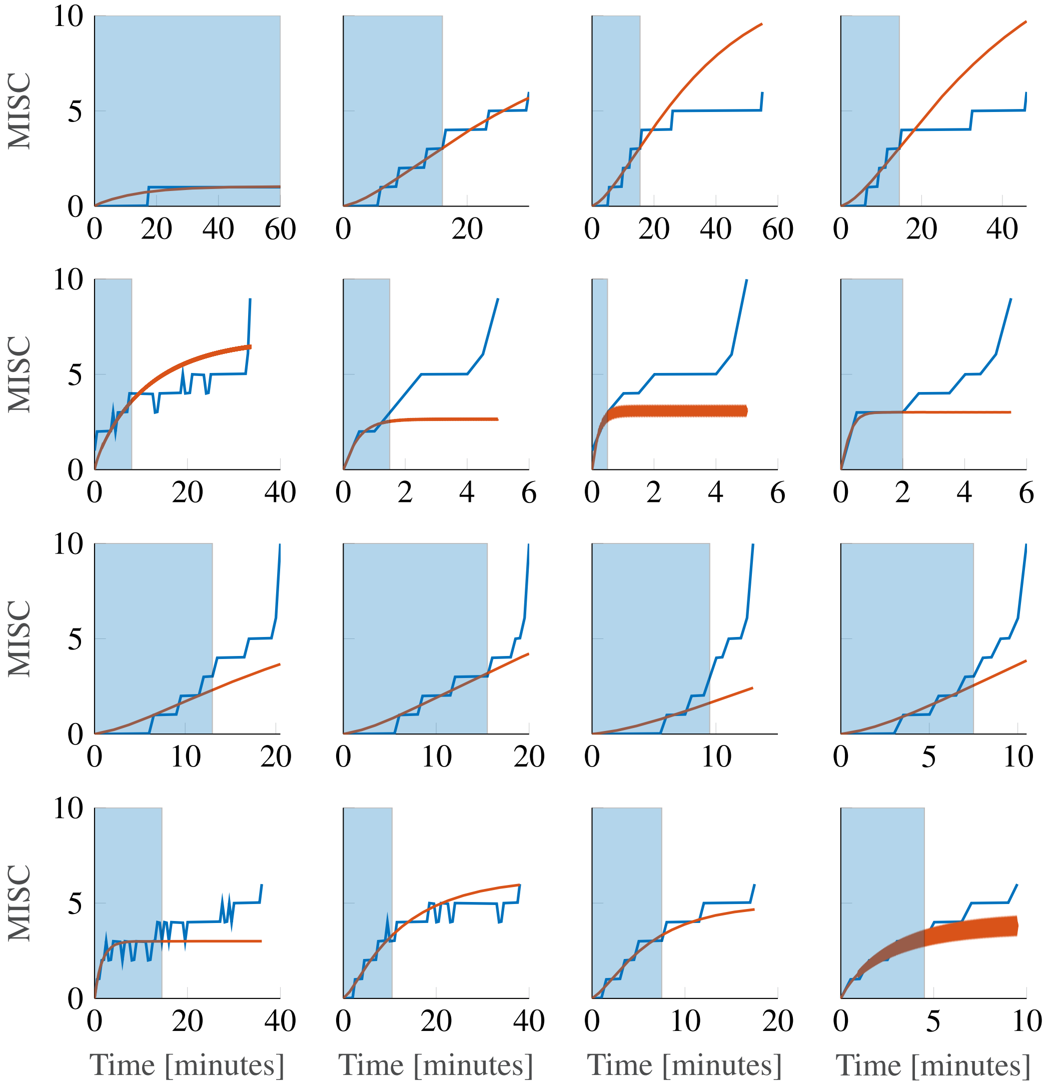

(c)

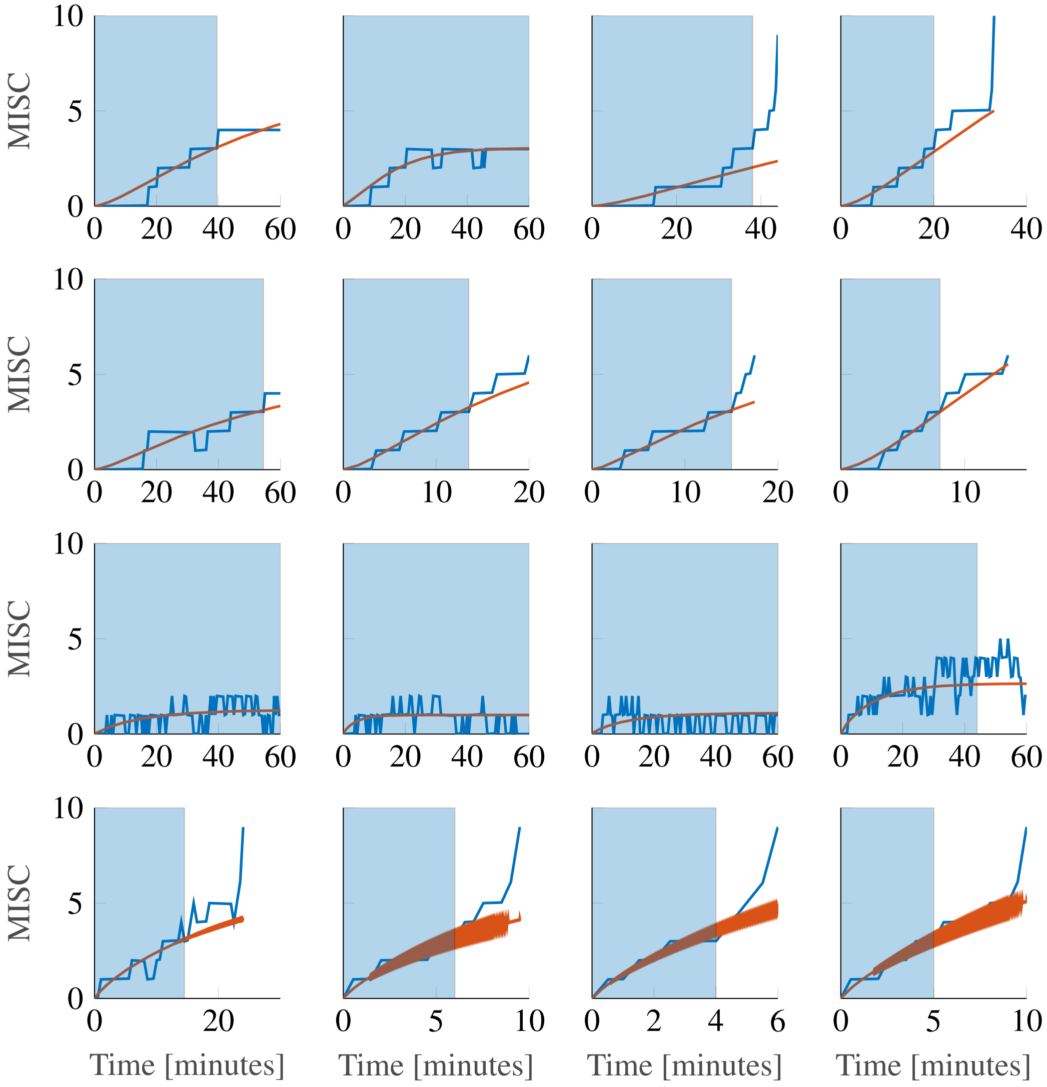

(d)

Figure S3: Extrapolations from MISC 3 to the end of the first motion phase, for the Oman model (in orange) and real observation (in blue) for all participants. The columns show responses for each amplitude condition, increasing in magnitude from left to right. The rows show results for each participant. The blue shaded area gives the observations the model has access to make forecasts
